# miR-16 and miR-103 impact 5-HT_4_ receptor signalling and correlate with symptom profile in irritable bowel syndrome

**DOI:** 10.1038/s41598-017-13982-0

**Published:** 2017-10-31

**Authors:** Carolin Wohlfarth, Stefanie Schmitteckert, Janina D. Härtle, Lesley A. Houghton, Harsh Dweep, Marina Fortea, Ghazaleh Assadi, Alexander Braun, Tanja Mederer, Sarina Pöhner, Philip P. Becker, Christine Fischer, Martin Granzow, Hubert Mönnikes, Emeran A. Mayer, Gregory Sayuk, Guy Boeckxstaens, Mira M. Wouters, Magnus Simrén, Greger Lindberg, Bodil Ohlsson, Peter Thelin Schmidt, Aldona Dlugosz, Lars Agreus, Anna Andreasson, Mauro D’Amato, Barbara Burwinkel, Justo Lorenzo Bermejo, Ralph Röth, Felix Lasitschka, Maria Vicario, Marco Metzger, Javier Santos, Gudrun A. Rappold, Cristina Martinez, Beate Niesler

**Affiliations:** 1 Department of Human Molecular Genetics, Institute of Human Genetics, University of Heidelberg, 69120 Heidelberg, Germany; 2 University of Leeds, St. James’s University Hospital, LS97TF Leeds, UK; 3 Mayo Clinic, Jacksonville, FL 32224 USA; 4 Medical Research Centre, Medical Faculty of Mannheim, University of Heidelberg, Mannheim, 68167 Germany; 5 Division of Bioinformatics and Biostatistics, National Centre for Toxicological Research, U.S. Food and Drug Administration (FDA), Jefferson, AR 72079 USA; 6 Digestive System Research Unit, Institut de Recerca Vall d’Hebron, Hospital Universitari Vall d’Hebron, Universitat Autònoma de Barcelona (Facultat de Medicina), 08035 Barcelona, Spain; 7 Department of Biosciences and Nutrition, Karolinska Institutet, 17177 Stockholm, Sweden; 8 Institute of Human Genetics, University of Heidelberg, 69120 Heidelberg, Germany; 9 Martin-Luther-Hospital, 14193 Berlin-Grunewald, Germany; 10 Oppenheimer Centre for Neurobiology of Stress, Division of Digestive Diseases, University of California, Los Angeles, CA 90095-7378 USA; 11 Washington University School of Medicine, St. Louis, MO 63110 USA; 12 TARGID, University Hospital Leuven, 3000 Leuven, Belgium; 13 Department of Internal Medicine & Clinical Nutrition, Institute of Medicine, Sahlgrenska Academy, University of Gothenburg, 41345 Gothenburg, Sweden; 14 Department of Medicine, Division of Gastroenterology and Hepatology, Karolinska University Hospital, Karolinska Institutet, Huddinge, 17176 Stockholm, Sweden; 15 Department of Clinical Sciences, Division of Internal Medicine, Skåne University Hospital, Malmö, Lund University, 22241 Lund, Sweden; 16 Department of Medicine, Division of Gastroenterology and Hepatology, Karolinska University Hospital, Karolinska Institutet, 14186 Stockholm, Sweden; 17 Division for Family Medicine and Primary Care, Karolinska Institutet, 14183 Huddinge, Sweden; 18 Department of Medicine, Solna, Karolinska Institutet, 171 76 Solna, Sweden; 19 Stress Research Institute, Stockholm University, 10691 Stockholm, Sweden; 20 Unit of Clinical Epidemiology, Department of Medicine, Karolinska Institutet, 171 76 Stockholm, Sweden; 21 BioDonostia Health Research Institute, San Sebastian and Ikerbasque, Basque Science Foundation, 48013 Bilbao, Spain; 22 Molecular Epidemiology Group, German Cancer Research Centre (DKFZ), Heidelberg, Germany; 23 Division of Molecular Biology of Breast Cancer, Department of Gynaecology and Obstetrics, University Women’s Clinic, University of Heidelberg, 69120 Heidelberg, Germany; 24 Institute of Medical Biometry and Informatics, University of Heidelberg, 69120 Heidelberg, Germany; 25 nCounter Core Facility, Institute of Human Genetics, University of Heidelberg, 69120 Heidelberg, Germany; 26 Institute of Pathology, University of Heidelberg, 69120 Heidelberg, Germany; 27 Department Tissue Engineering and Regenerative Medicine (TERM), University Hospital Wuerzburg, 97082 Wuerzburg, Germany; 28 Translational Centre ‘Regenerative Therapies for Oncology and Musculoskeletal Diseases’ (TZKME), Branch of the Fraunhofer Institute Interfacial Engineering and Biotechnology (IGB) Wuerzburg, 97082 Wuerzburg, Germany

## Abstract

Irritable bowel syndrome (IBS) is a gut-brain disorder involving alterations in intestinal sensitivity and motility. Serotonin 5-HT_4_ receptors are promising candidates in IBS pathophysiology since they regulate gut motor function and stool consistency, and targeted 5-HT_4_R selective drug intervention has been proven beneficial in subgroups of patients. We identified a single nucleotide polymorphism (SNP) (rs201253747) c.*61 T > C within the 5-HT_4_ receptor gene *HTR4* to be predominantly present in diarrhoea-IBS patients (IBS-D). It affects a binding site for the miR-16 family and miR-103/miR-107 within the isoforms *HTR4b/i* and putatively impairs *HTR4* expression. Subsequent miRNA-profiling revealed downregulation of miR-16 and miR-103 in the jejunum of IBS-D patients correlating with symptoms. *In vitro* assays confirmed expression regulation via three 3′UTR binding sites. The novel isoform *HTR4b_2* lacking two of the three miRNA binding sites escapes miR-16/103/107 regulation in SNP carriers. We provide the first evidence that *HTR4* expression is fine-tuned by miRNAs, and that this regulation is impaired either by the SNP c.*61 T > C or by diminished levels of miR-16 and miR-103 suggesting that *HTR4* might be involved in the development of IBS-D.

## Introduction

Irritable bowel syndrome (IBS) is a common gastrointestinal (GI) disorder affecting over 15% of the population worldwide. The burden of illness of IBS is significant, and includes considerable impact on quality of life and work productivity of affected individuals^1^.

The syndrome is currently defined by chronically recurring abdominal pain and altered bowel habits. Patients are subclassified into either IBS with diarrhoea (IBS-D), with constipation (IBS-C), mixed bowel habit (IBS-M) or unsubtyped IBS^2^.

The aetiology and pathophysiology of IBS is multifactorial including genetic and environmental factors^3–5^. The most substantial findings to date have been reported for the serotonergic and immune system as well as neuronal and gut epithelial barrier function^6^.

Over the last decade, converging evidence has implicated the serotonergic system as a key player in the pathophysiology of IBS. Serotonin (5-hydroxytryptamine, 5-HT) is released from enterochromaffin (EC) cells^7^. 5-HT regulates various processes including gut motility, secretion, visceral sensation and neuronal signalling within the brain-gut axis which are often impaired in IBS patients. Moreover, perturbances in plasma levels of 5-HT, changes in expression of key components of the 5-HT system, such as the serotonin transporter (SERT) and tryptophan hydroxylase have been observed^8^.

5-HT exerts different functions via interaction with a variety of 5-HT receptors in the gut, which reside on neurons, epithelial and smooth muscle cells. Relevant to this work, 5-HT_4_ receptors are broadly expressed in the human intestine^9,10^ and can be found on inhibitory nitrergic neurons to induce smooth muscle relaxation and on cholinergic neurons to control muscle contraction. In addition to their neuronal localization, they reside on enterocytes and enteroendocrine cells of the gut mucosa, regulating the secretion of fluid, mucus and 5-HT^10,11^.

Based on their wide range of functions within the intestine, 5-HT_4_ receptors are attractive targets for IBS therapy. 5-HT_4_ receptor agonists benefit patients with IBS-C and functional constipation by increasing motility and accelerating transit^12^.

**Figure 1:**
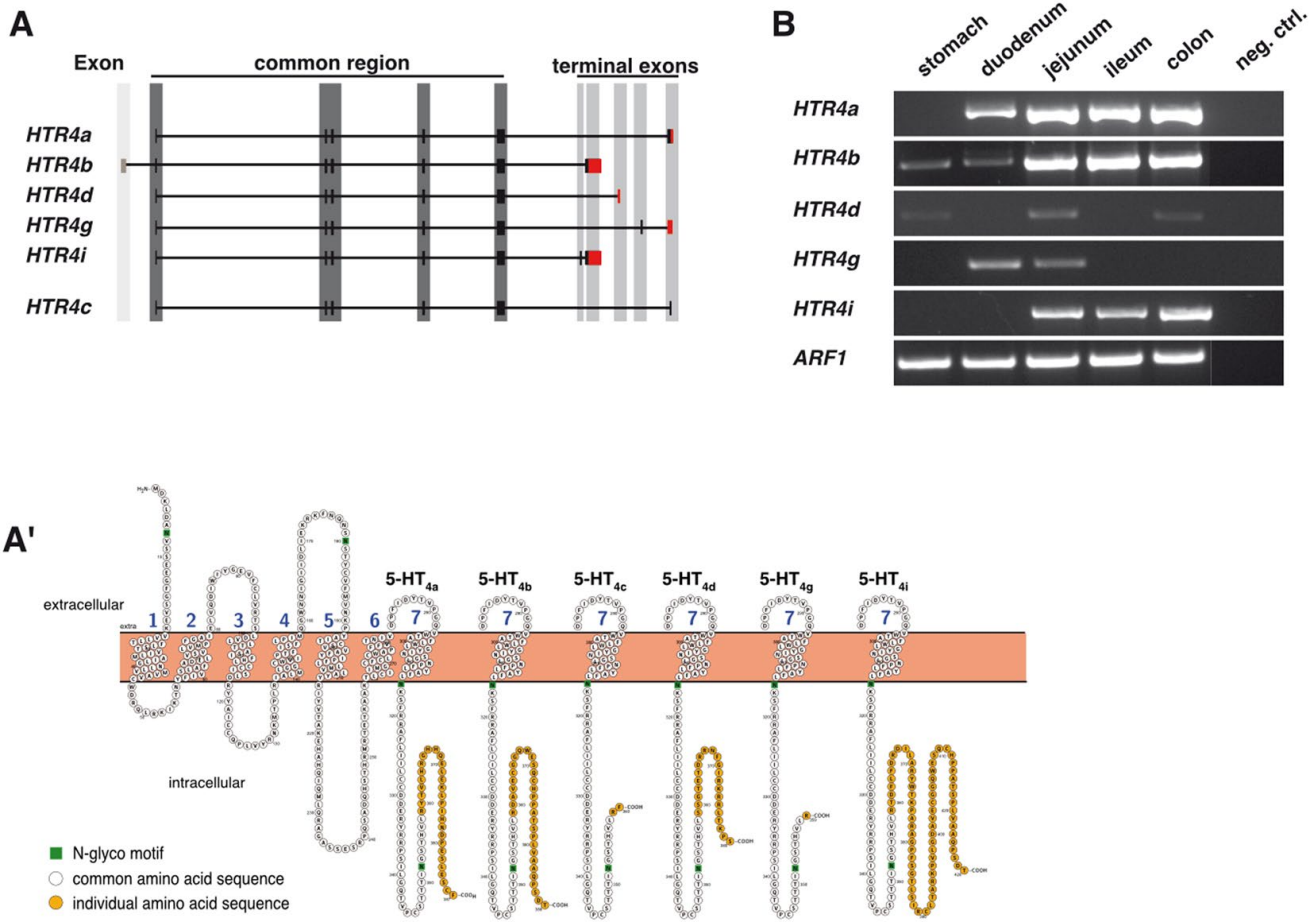
Illustration of *HTR4* isoforms and their expression pattern in the human GI tract. (**A**) *HTR4* isoforms *HTR4a*, *HTR4b*, *HTR4d*, *HTR4g*, *HTR4i* and *HTR4c*, without a specified 3′UTR. The common region (dark grey) encompasses exons 2-6 and is shared by all six *HTR4* isoforms. The isoform-specific 3′UTRs are highlighted in red and encode unique 5-HT_4_R C-termini. Not drawn to scale. (**A′**) 5-HT_4_ receptor isoform transmembrane topologies indicating individual C-terminal ends predicted by *Protter* for visualization of proteoforms (http://wlab.ethz.ch/protter). (**B)** Expression pattern of *HTR4* isoforms in different human GI regions. *ARF1* served as mRNA integrity and loading control. Respective PCR images were cropped for figure implementation.

Currently, six isoforms of the human 5-HT_4_ receptor gene *HTR4* are annotated in the NCBI GenBank. The majority of the remaining isoforms have not yet been determined. Besides a common region at their N-terminal end until position L358, their amino acid sequences differ at their C-terminal end (Fig. 1A′). All isoforms mainly vary on mRNA level presenting with individual 3′ untranslated regions (3′UTR) (Fig. 1A). These regions represent the major site of microRNA (miRNA, miR) interaction and post-transcriptional regulation.

Mature miRNAs are small single stranded non-coding RNAs of 18–25 nucleotides. For target recognition, they depend on a roughly seven nucleotides spanning seed sequence at their 5′ end being perfectly complementary to the respective mRNA^13^. Since the sequence complementarities and the thermodynamics of binding play an essential role in the interaction of miRNA with its target mRNA, sequence variations in the miRNA-binding seed regions are prone to reinforce, weaken, or disrupt the miRNA-mRNA interactions and thereby to affect the expression of mRNA targets. This impairment contributes fundamentally to disease aetiology^14^.

To date, disturbed regulation of seven miRNAs and their targets has been implicated in IBS^15–20^. The main focus of the current study was to determine whether miRNAs play a role in the differential expression regulation of the 5-HT_4_ receptor gene in IBS.

Moreover, we hypothesized that dysregulation of *HTR4* expression by miRNAs either due to polymorphic target sites or differential miRNA expression profiles might contribute to the pathophysiology of IBS. Our specific aims were to locate SNPs residing within the 3′UTRs of GI-relevant *HTR4* isoforms in order to identify putatively regulating miRNAs and to assess miRNA expression in gut biopsies of IBS patients compared to healthy controls and to validate the regulation *in vitro*.

## Results

### Distinct expression of *HTR4* isoforms in the human GI tract

Initially, the expression profiles of the five as yet annotated isoforms of the *HTR4* gene in NCBI GenBank (*HTR4a* NM_001040169.2, *HTR4b* NM_000870.5, *HTR4d* NM_001040172.2, *HTR4g* NM_199453.3 and *HTR4i* NM_001040173.2) were assessed. Mandatory to mention for subsequent analyses, all isoforms differ at their 3′ ends, except *HTR4b* and *HTR4i* with identical 3′UTRs (Fig. 1A). At the protein level, all annotated isoforms share a common region at their N-terminal end to position L358, whereas their amino acid sequences differ at the C-terminus (Fig. 1A′). Expression analyses using total RNA from normal stomach, duodenum, jejunum, ileum and colon revealed considerably higher expression levels of *HTR4a*, *HTR4b* and *HTR4i* in distal small bowel and colon compared to the proximal intestine (Fig. 1B). Isoforms *HTR4a* and *HTR4i* were not expressed in the stomach and *HTR4i* was also absent in the duodenum. *HTR4d* and *HTR4g* showed a weaker and distinct expression pattern suggesting a minor role in the gut (Fig. 1B).

### *HTR4* c.*61 T > C resides in a putative miRNA binding site in the 3′UTR of *HTR4b/i* and is associated with IBS-D

**Figure 2:**
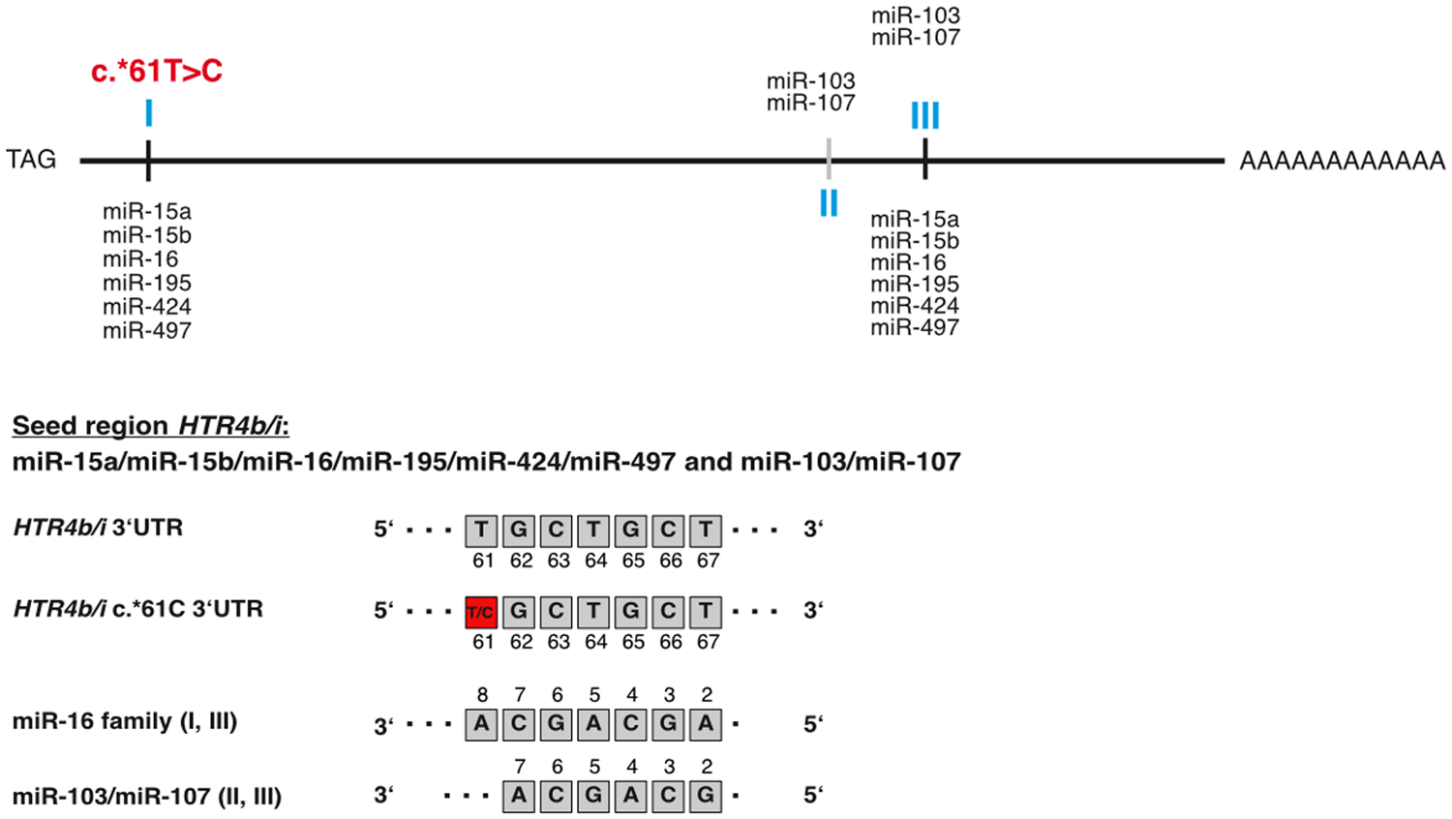
Schematic illustration of the miR-16 family and miR-103/miR-107 binding sites in the 3′UTR of the *HTR4b/i* isoforms. Binding sites (I-III) are based on predictions by TargetScan, miRanda and/or RegRNA. The position of *HTR4b/i* c.*61 T > C is highlighted in red within the seed region (nucleotides 2–8) of the indicated miRNA binding site.

3′UTRs represent the major binding sites for miRNA-dependent post-transcriptional regulation. In order to unravel a putative interplay between miRNAs and the *HTR4* 3′UTRs, we first assessed the genetic heterogeneity of isoform-specific untranslated regions. Moreover, as it is well established that functionally relevant SNPs reside in 3′UTRs near the stop codons, we exclusively screened the stop codon surrounding regions of the GI-relevant *HTR4* isoforms *HTR4a, b* and *i*. First, we analysed a discovery sample from the UK and detected a rare polymorphism (c.*61 T > C; rs201253747) in two out of 98 screened IBS-D patients in a heterozygous manner. This SNP locates in the 3′UTRs of the *HTR4b* and *HTR4i* isoforms, which both share the identical 3′UTR (Fig. 1A). None of the other clinically defined individuals, neither the controls nor IBS-C patients, carried this variant. miRNA target site analysis of the *HTR4b/i* 3′UTR by *in silico* prediction tools (TargetScan, miRanda and/or RegRNA) suggested that the variant locates within the heptameric seed region (*ACGACGA*) of a putative binding site (Fig. 2; indicated by I-III) for the miR-16 family (I: including miR-15a (hsa-mir-15a; MI0000069), miR-15b (hsa-mir-15b; MI0000438), miR-16 (hsa-mir-16-1; MI0000070/hsa-mir-16-2; MI0000115), miR-195 (hsa-mir-195; MI0000489), miR-424 (hsa-mir-424; MI0001446), miR-497 (hsa-mir-497; MI0003138)). Moreover, additional putative binding sites for miR-103/miR-107 (II: hsa-mir-103-1; MI0000109/hsa-mir-103-2; MI0000108/hsa-mir-107; MI0000114) alone and combined with the miR-16 family (III) were identified further downstream in the *HTR4b/i* 3′UTRs, all sharing a hexameric, shortened *ACGACG* seed sequence (see above) (Fig. 2). A more comprehensive analysis taking all *HTR4* isoforms into account confirmed binding sites for miR-16 and miR-103/miR-107 in the GI-relevant isoforms *HTR4b* and *HTR4i*. However, this analysis also revealed binding sites in *HTR4g*. Since this isoform was only found to be weakly expressed in the duodenum and jejunum, it was not further taken into consideration (Fig. 1B).

To replicate our initial finding, we genotyped five additional cohorts from Germany, Belgium, Sweden and the USA (EAM, GS) and performed a pooled analysis (in total 832 IBS-D and 614 IBS-C patients as well as 2273 healthy controls, Supplementary Table S2). Thereby, we confirmed the variant c.*61 T > C to be significantly more frequent in IBS-D patients compared with healthy controls and all non-IBS-D patients (p = 0.049, OR = 2.74 (95% CI = 0.961-7.854) and p = 0.039, OR = 2.71 (95% CI = 1.007-7.308) respectively, Table 1). No deviation from the Hardy-Weinberg Equilibrium (HWE) was detected in the IBS patients or healthy controls.

**Table 1:** *HTR4b* c.*61 T > C (rs201253747) genotypes in IBS patients and healthy controls

Genotype	c.*61 T/c.*61 T	c.*61 T/ c.*61 C	c.*61 C/c.*61 C	MAF (C)	P-value/OR/95% CI
IBS-D	825	7	0	0.00420	
IBS-C	612	2	0	0.00163	
controls	2266	7	0	0.00154	0.049/2.74/0.961-7.854 (IBS-D vs. controls)
non-IBS-D	2878	9	0	0.00156	0.039/2.71/1.007-7.308 (IBS-D vs. non-IBS-D)

IBS, Irritable bowel syndrome; D, diarrhoea; C, constipation; MAF, minor allele frequency; OR, odds ratio; CI, confidence interval.

### Identification of two novel alternatively spliced *HTR4b* 3′UTR isoforms

Since the functional relevance of the *HTR4b/i* c.*61 C variant with IBS-D remained elusive at this stage, we analysed the *HTR4b/i* 3′UTR for alternatively spliced or polyadenylated isoforms, as literature provided evidence for more than six *HTR4* isoforms (Fig. 1A). Currently 25 human isoforms can be found in the NCBI GenBank, mostly varying in the composition of their 3′UTRs^21^.

**Figure 3:**
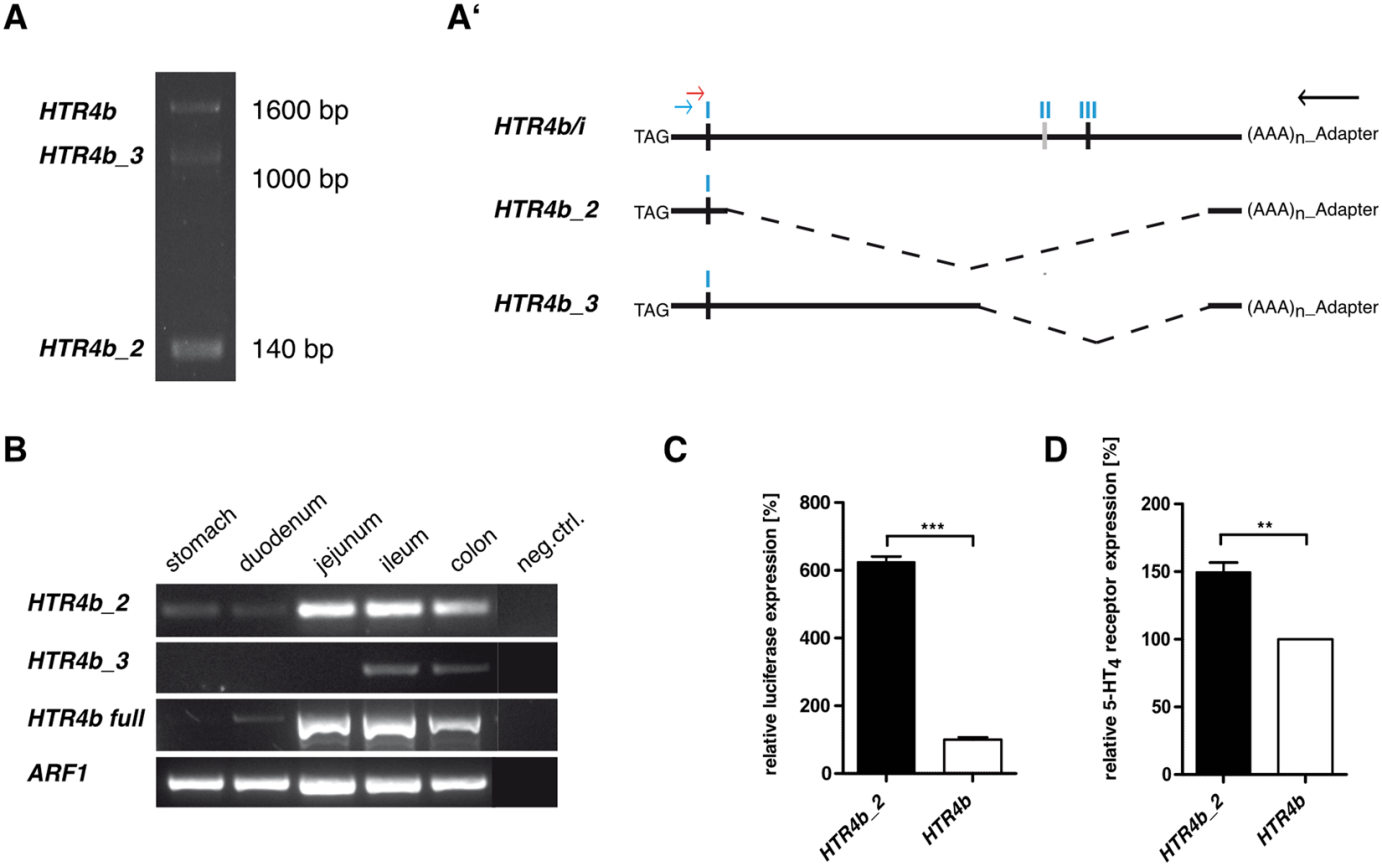
Identification of two novel *HTR4b* splice variants by 3′RACE. **(A)** Three different isoforms were identified by 3′RACE. **(A′)** Schematic illustration of the 3′UTRs of full length *HTR4b* and the novel isoforms *HTR4b_2* and *HTR4b_3*. miRNA binding sites are indicated by ‘I-III’ (in blue). Arrows reflect positions of 3′RACE primers. (**B**) Expression pattern of the three *HTR4b* isoforms in different human GI tissues. *ARF1* served as cDNA integrity and loading control. Respective PCR images were cropped for figure implementation. (**C**) Relative luciferase activity of *HTR4b_2* and *HTR4b* 3′UTR reporter gene constructs (n = 3) and (**D**) Relative 5-HT_4b_ and _b2_ receptor levels quantified by In Cell Western experiments (n = 3) in HEK293T. Values are means ± SEM., **p < 0.01, ***p < 0.001. Unpaired t-test.

Therefore, 3′RACE (rapid amplification of cDNA ends) experiments were carried out using total RNA from human ileum and colon. Three major amplicons of different size were generated and cloned for sequence verification (Fig. 3A). All three amplicons were confirmed by RT-PCR and Sanger sequencing to represent novel isoforms of *HTR4b* (Fig. 3 - Supplementary Figure S1). Besides the established *HTR4b* isoform, two shorter versions of the canonical full length *HTR4b* 3′UTR with splicing sites at positions 1414/2903 (*HTR4b_2*; NCBI GenBank accession number BankIt2040902 HTR4b_2MF775735) and 2197/2903 (*HTR4b_3*; NCBI GenBank accession number BankIt2040902 HTR4b_3MF775736) were confirmed (positions correlate to NM_000870.5). Both novel isoforms lack the miR-103/miR-107 (II) binding site and the highly regulatory region with the double miR-16 family/miR-103/107 binding site (III), resulting in one remaining target site for the miR-16 family (I; Fig. 3A′). Subsequent RT-PCR analysis showed a fairly ubiquitous expression pattern of *HTR4b_2* in the investigated GI tissue regions (Fig. 3B). Expression of *HTR4b_3* was very weak, restricted to ileum and colon and therefore not further investigated (Fig. 3B). Luciferase reporter assays (Fig. 3C) and In Cell Western (Fig. 3D) experiments in HEK293T cells showed significantly higher luciferase activity and protein expression level for the shortened *HTR4b_2* isoform compared to the full length *HTR4b* isoform (Fig. 3C and D), indicating that *HTR4b_2* might be more efficiently translated into protein.

### The miR-16 family is co-expressed with *HTR4b* and *HTR4b_2* in different subregions of the human colon

**Figure 4:**
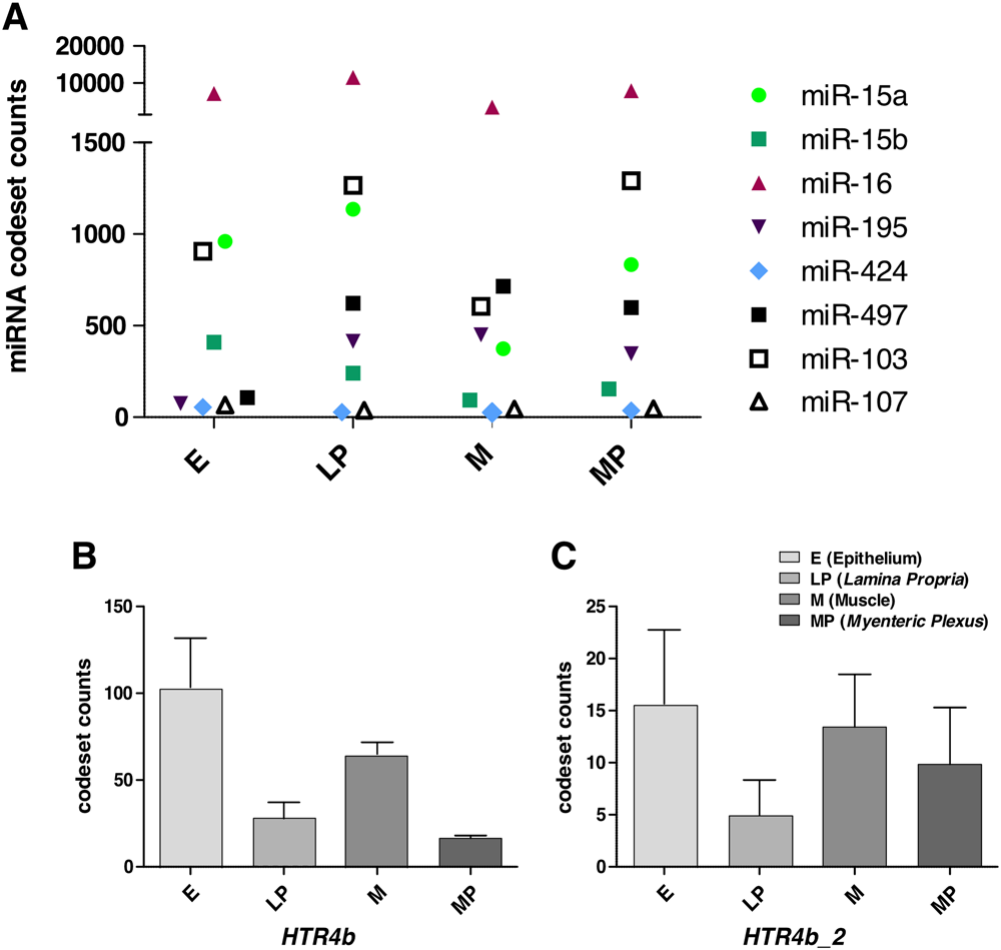
Expression analyses of relevant miRNAs, *HTR4b and HTR4b_2* in human colonic subregions. nCounter miRNA expression profile of (**A)** selected miR-16 family members as well as of miR-103/miR-107. (**B)** *HTR4b* and (**C)** *HTR4b_2* in normal laser capture microdissected human colonic subregions. Values are means ± SEM of codeset counts from total RNA of tissue specimens from four individuals, respectively. E (epithelium), LP (*lamina propria*), M (muscle), MP (*myenteric plexus*).

Previous to the evaluation of the functional impact of miRNAs on *HTR4* isoform expression levels, we had to ascertain that respective miRNAs and *HTR4* isoforms are subregionally co-expressed. In doing so, distinct miRNA expression was assessed in colonic subregions from normal microdissected tissue (epithelium, *lamina propria*, muscle and *myenteric plexus*) by the RNA quantification technology nCounter. In order to elucidate overlapping expression patterns with the putative miRNA targets *HTR4b, HTR4b_2*, and *HTR4i*, we analysed corresponding material with isoform-specific detection probes. Initially, layer specificity was proven by cell type-specific marker assessment (data not shown). Except miR-424 and miR-107, all members of the miR-16 family as well as miR-103 were detectable in each of the tested colonic subregions in moderate to high levels with miR-16 and miR-103 showing peak levels (Fig. 4A). In addition, *HTR4b* (Fig. 4B) and *HTR4b_2* (Fig. 4C) were expressed in all subregions analysed, showing the lowest expression levels in the *lamina propria* and in the *myenteric plexus* (only for *HTR4b*). In contrast, *HTR4i* seems to be primarily expressed in the *lamina propria* and, to a lower extent, in the other investigated layers (Fig. 4 - Supplementary Figure S2). Based on the differential expression of *HTR4i* we focused in subsequent approaches on *HTR4b* and *HTR4b_2* only.

### Expression of *HTR4b* is downregulated by the miR-16 family and by miR-103 *in vitro*

**Figure 5:**
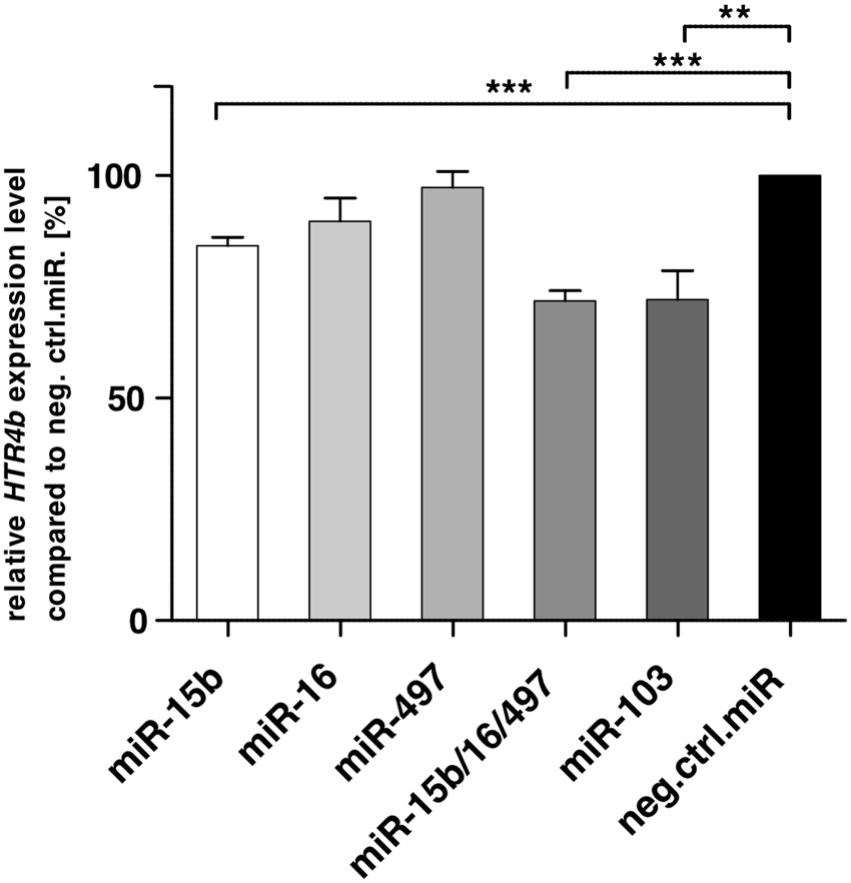
*HTR4b* mRNA levels after overexpression of several miRNAs in Colo320 cells analysed by qPCR. Relative expression analysis of *HTR4b* mRNA levels after transfection (72 h) with different miRNAs (miR-15b, miR-16, miR-497, miR-15b/16/497, miR-103) and a negative control miR (neg. ctrl. miR). Values are means ± SEM of three to four independent experiments and were normalised to *SDHA*. *p < 0.05; ***p < 0.001. Unpaired t-test.

To investigate the functional impact of the relevant miRNAs on the expression levels of *HTR4b*, we transfected the respective miRNA precursor molecules, either separately or combined, into colon adenocarcinoma Colo320 cells and quantified *HTR4b* transcript levels by qPCR. The largest and most significant reduction of the *HTR4b* mRNA level was observed in cells transfected with a combination of the miR-16 family members miR-15b/16/497 as well as with miR-103 (Fig. 5). In contrast, overexpression of individual members of the miR-16 family (miR-15b, miR-16 and miR-497) resulted in minor mRNA changes (Fig. 5).

### The *HTR4b*_2 novel isoform carrying the c.*61 T > C SNP escapes miRNA regulation

**Figure 6:**
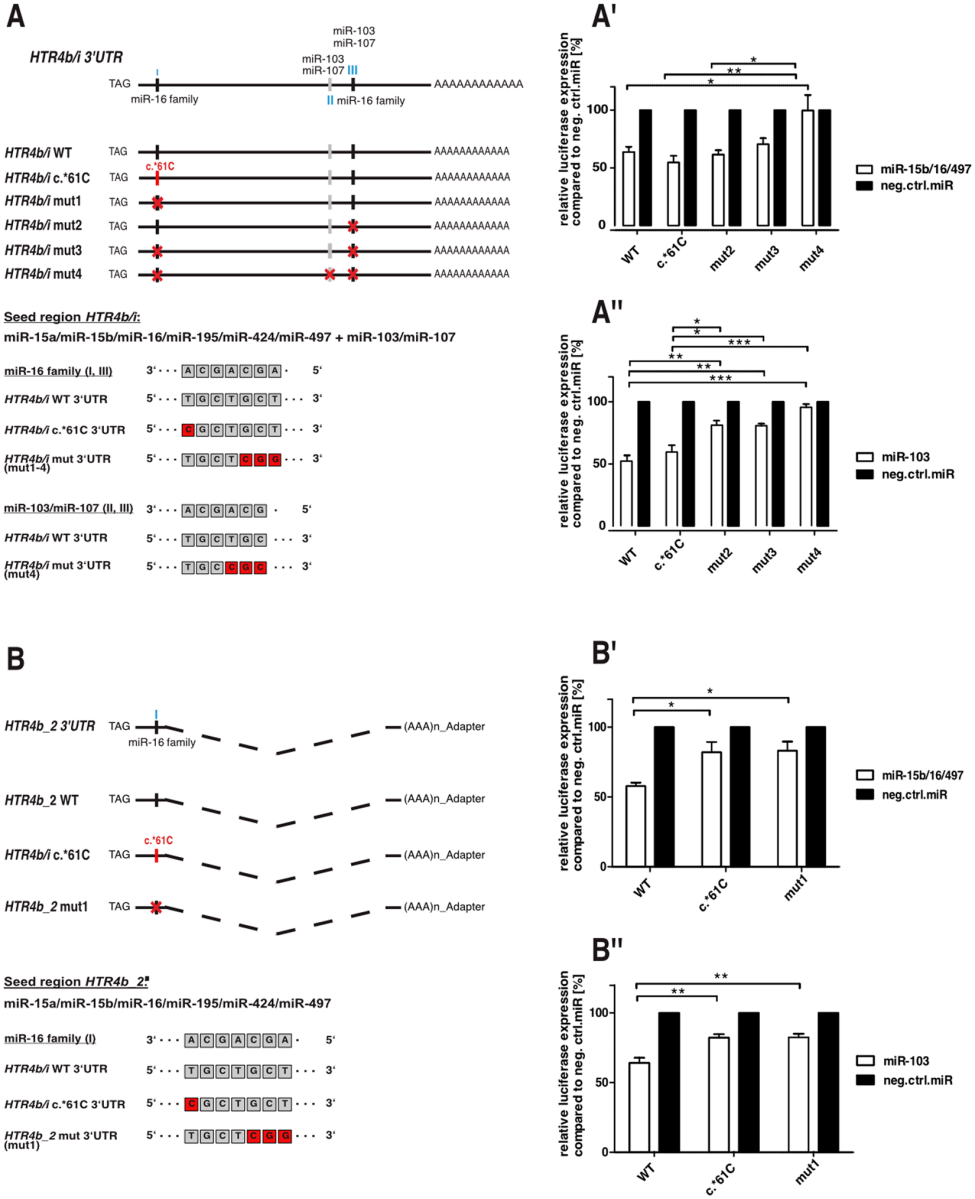
The *HTR4b*_2 novel isoform carrying the c.*61 T > C SNP escapes miRNA regulation. Illustration of the *HTR4b/b_2* related luciferase reporter gene expression experiments. (**A)** Respective *HTR4b/i* 3′UTRs including predicted miRNA target sites (I-III, indicated in blue) and luciferase gene reporter constructs. (**A′)** Relative luciferase activity in HEK293T transfected with miR-16 family members. (**A″)** Relative luciferase activity in Colo320 transfected with miR-103. **(A′)** and **(A″)** Particular miRNAs are both co-expressed with respective luciferase constructs (WT, c.*61 C, mut2, mut3, mut4) and related to negative control miRNA (neg. ctrl. miR). Values are means ± SEM. n = 4 **(A′)** and n = 3 **(A″)** experiments for each condition. **(B)** Respective *HTR4b_2* 3′UTR including predicted miRNA target site (I) and luciferase gene reporter constructs. **(B′)** Relative luciferase activity in Colo320 transfected with miR-15b/16/497 (miR-16 family) or **(B″)** relative luciferase activity in Colo320 transfected with miR-103. (**B′)** and **(B″)** Particular miRNAs are both co-expressed with luciferase constructs (WT, c.*61 C, mut1) or negative control miRNA (neg. ctrl. miR). Values are means ± SEM. (n = 5) **(B′)** and (n = 4) **(B″)** experiments for each condition. *p < 0.05; **p < 0.01; ***p < 0.001. One-way ANOVA with Bonferroni post-hoc test, WT (wild type), mut (mutated).

As evidence accumulated that respective *HTR4* isoforms are co-expressed with and transcriptionally regulated by relevant miRNAs, we proceeded by assessing the importance of distinct miRNA target sites, and in particular the functional relevance of the identified rare variant (c.*61 T > C) associated with IBS-D. For this purpose we performed luciferase reporter assays with the canonical *HTR4b/i* isoforms by cloning both, the full length wild type (WT) *HTR4b/i* 3′UTR and the corresponding mutated construct (c.*61 C). Moreover, we created four additional mutated (mut) 3′UTR *HTR4b* constructs designed to disrupt each of the three miRNA binding sites (I-III) individually and all three at once (Fig. 6A).

A combination of miR-15b/16/497 significantly decreased reporter expression of the WT construct (Fig. 6A′). However, this effect could not be rescued by the *HTR4b* c.*61 C variant nor by the disrupted first (I; mut1) or second (III; mut2) miR-16 family binding site. A significant increase of reporter levels was only accomplished by the simultaneous disruption of both (I, III; mut3) miR-16 family target sites (Fig. 6A′). Corresponding experiments co-expressing luciferase constructs with miR-103 showed a similar pattern as WT and *HTR4b* c.*61 C. Reporter levels were both significantly reduced (Fig. 6A″). In addition, the simultaneous disruption of the second (II; mut2) and third (III; mut4) target site led to an incremental rescue of reporter expression (Fig. 6A″).

The novel isoform *HTR4b_2* only harbours the miRNA binding site (I: miR-16 family, Fig. 6B) where the c.*61 T > C SNP resides. Therefore, it was our aim to assess the functional impact of the SNP on *HTR4b_2* expression regulation. As shown in Fig. 6B′, the co-expression with miR-15b/16/497 reduced the *HTR4b_2* WT reporter levels to a significant extent. Remarkably, this effect was counteracted by both the *HTR4b_2* c.*61 C variant and the completely disrupted miRNA binding site (I; mut1) (Fig. 6B′). Binding of miR-103 to this particular target site was not predicted by computational tools. However, as miR-103 shares six of the seven seed nucleotides with the miR-16 family (Fig. 6A), we assumed that it could additionally affect *HTR4b_2* expression. Indeed, when co-expressed with miR-103, the WT reporter signal was significantly reduced (Fig. 6B″). The presence of either the *HTR4b* c.*61 C variant or the disrupted miRNA binding site (I; mut1) significantly weakened this regulation (Fig. 6B″).

### The mutated miR-16 A > G attenuates the regulation of *HTR4b_2* through miR-16 in human goblet cells

We next aimed to gain additional evidence for the functional relevance of miRNA regulation on the *HTR4b/i* c.*61 T > C variant and the novel *HTR4b*_2 isoform. As gut biopsies of SNP carriers were not available, we applied a complementary approach to investigate non-cancer cell lines. Since the expression and functional role of 5-HT_4_ receptors in goblet cells had previously been elucidated^10^, we used the human colonic goblet cell line HT29-MTX-E12. In order to inversely mimic the *in vivo* situation of SNP carriers, we applied a mutated miR-16 precursor carrying the corresponding mutation within the seed region (hsa-miR-16 A > G, Supplementary Figure S3A). Goblet cells showed only marginal levels of *HTR4b*, while the shorter isoform *HTR4b_2* was robustly expressed (Supplementary Figure S3B); therefore, we restricted the following analyses to the *HTR4b_2* isoform. miR-16 induced a significant reduction of *HTR4b_2* expression compared to the negative control miR. In contrast, an increased *HTR4b_2* expression by both miR-16 A > G, mimicking the SNP situation, and miR-103, was observed although differences did not reach statistical significance (Supplementary Figure S3C).

### miR-16 and miR-103 are significantly downregulated in the small intestine of IBS-D patients

**Figure 7:**
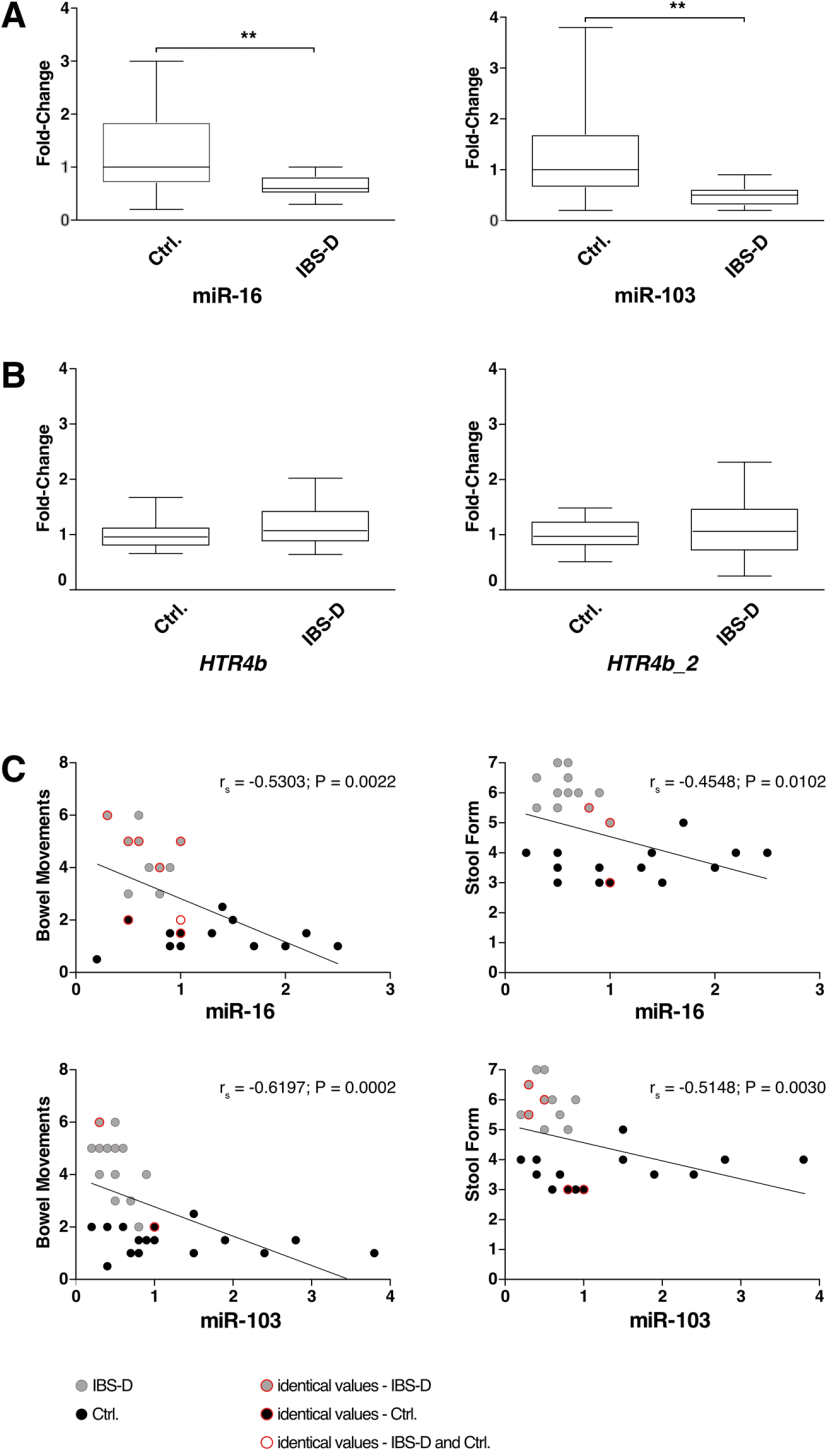
Expression analysis of miR-16 and miR-103 and *HTR4b* and *HTR4b_2* in the human jejunal mucosa in IBS-D vs. healthy controls (Ctrl.). (**A**) qPCR analysis of miR-16 and miR-103. Fold-change value is based on the ratio of target miRNA and the average of reference genes normalised to the average of the healthy group. Values are means ± SEM. (14 IBS-D; 17 controls). **p < 0.01. Unpaired t-test with Welch’s correction. (**B**) nCounter analyses of *HTR4b* and *HTR4b_2*. Fold-change is based on the ratio between target mRNA and the average of the reference genes normalised to the average of the healthy control group. Values are means ± SEM. (30 IBS-D; 18 controls). Mann-Whitney *U* test. (**C**) Correlations of IBS symptoms with miRNA expression in the jejunal mucosa of IBS-D patients and controls. Spearman’s correlation rho was applied to the pooled data. Identical values refer to multiple equal correlation values of different individuals. n = 31 (14 IBS-D; 17 controls). Spearman’s rho (r_s_) and p-values (p) are given.

To further understand the role of the GI subregionally most abundant miR-16 and miR-103 in IBS, we performed comparative expression analysis by qPCR on intestinal biopsy samples from the jejunum of IBS-D patients compared to controls. This revealed a significant downregulation of miR-16 and miR-103 in IBS-D patients compared with healthy controls (Fig. 7A). To validate a putative regulation of both the canonical *HTR4b* and the novel *HTR4b*_2 isoform on mRNA level, we further assessed their expression by nCounter analysis in jejunal biopsies of patients and controls. However, no differences in expression levels were found on the mRNA level, neither for *HTR4b* nor *HTR4b_2* (Fig. 7B). In addition, genotyping of the tested samples did not identify any SNP carriers (data not shown). To the best of our knowledge and based on extensive analysis of available anti-5-HT_4_ antibodies, currently no 5-HT_4b_ specific antibody exists. Therefore, we were not able to confirm differential expression on the receptor protein level (see Supplementary information).

### Bowel movements and stool form of IBS-D patients correlate with miR-16 and miR-103 expression

To reveal the potential clinical relevance of our findings, we successively applied the Spearman’s correlation rho to pooled data of IBS-D patients and healthy controls taking bowel movements and stool form into consideration amongst others (Fig. 7C). This analysis showed that the expression of miR-16 and miR-103 negatively correlated with bowel habits (Spearman’s rho r_s_ and p-values are given in Fig. 7C).

## Discussion

In this study, we identified a regulatory SNP affecting a miRNA binding site and confirmed isoform-specific miRNA regulation, both relevant in IBS-D. Multiple target sites within the 3′UTR ensure miRNA-mediated expression control in a redundant manner and are able to compensate the mismatch caused by the minor *HTR4b/i* c.*61 C allele in the full length form. Our study revealed the novel short 3′UTR of *HTR4b_2* to be potently downregulated by miR-16 family members as well as miR-103 via the remaining miR-16 target site (I). In addition, we showed that the minor *HTR4b/i* allele c.*61 C impairs miRNA binding, thereby increasing reporter levels by 20-30% for *HTR4b_2*. This may in turn correspond to increased expression of the 5-HT_4b2_ receptor isoform in c.*61 C carriers. Since no gut biopsies of SNP carriers were available, we mimicked the *in vivo* situation by applying a mutated miR-16 precursor (miR-16 A > G) carrying the corresponding mutation in a human goblet cell line. Thereby, we were able to counteract the downregulation, pointing to the deregulation of 5-HT_4_ receptor expression fine-tuning in SNP carriers.

miR-16 family members are ubiquitously expressed in moderate to high levels, and at least one family member seems to be present in every human cell type pointing to their importance in various cellular processes^22^. In particular, miR-103 is most ubiquitously, whereas all other miR-16 family members are distinctly and moderately to marginally expressed in the GI tract^23^. Our data corroborates these findings showing moderate to high expression for all, except miR-424 and miR-107, with peak levels of miR-16 and miR-103 in the analysed colon layers. In contrast, *HTR4b* and *HTR4b_2* show a more distinct expression profile in the respective subregions compared to *HTR4i* pointing to more specific roles in the respective GI layers. The overlap in expression of *HTR4b/HTR4b_2* and the miR-16 family in the particular colonic subregions make the fine-tuning of 5-HT_4b/b_2_ receptor levels by miR-16 and miR-103 in the GI tract obvious.

To date, approximately 60% of all protein coding genes are estimated to be targeted by miRNAs and miRNA target site polymorphisms have been functionally linked to several diseases^24–26^. *HTR4b/i* c.*61 T > C is the second variant of a serotonin receptor gene associated with IBS-D since we identified the functional *HTR3E* variant c.*76 G > A^15^, both of which lead to disturbed miRNA regulation and potentially affect receptor densities.

Quantitative analysis of total 5-HT_4_ receptor levels within GI subregions in *HTR4b/i* c.*61 T > C carriers is mandatory for final proof. Yet SNP carriers are rare and currently no reliable 5-HT_4_ receptor antibodies suitable for quantitative Western blot analysis are available (see Supplementary information and Supplementary Figure S4), making such an approach challenging at present. As there are no animal and disease models in molecular genetics to study IBS, a functional follow up in an *in vivo* model is currently not feasible. We presently depend on human tissue material and *in vitro* test systems. The establishment of patient-derived primary cell culture models may help to further elucidate disease-associated pathways and mechanisms at the molecular level in future approaches.

**Figure 8:**
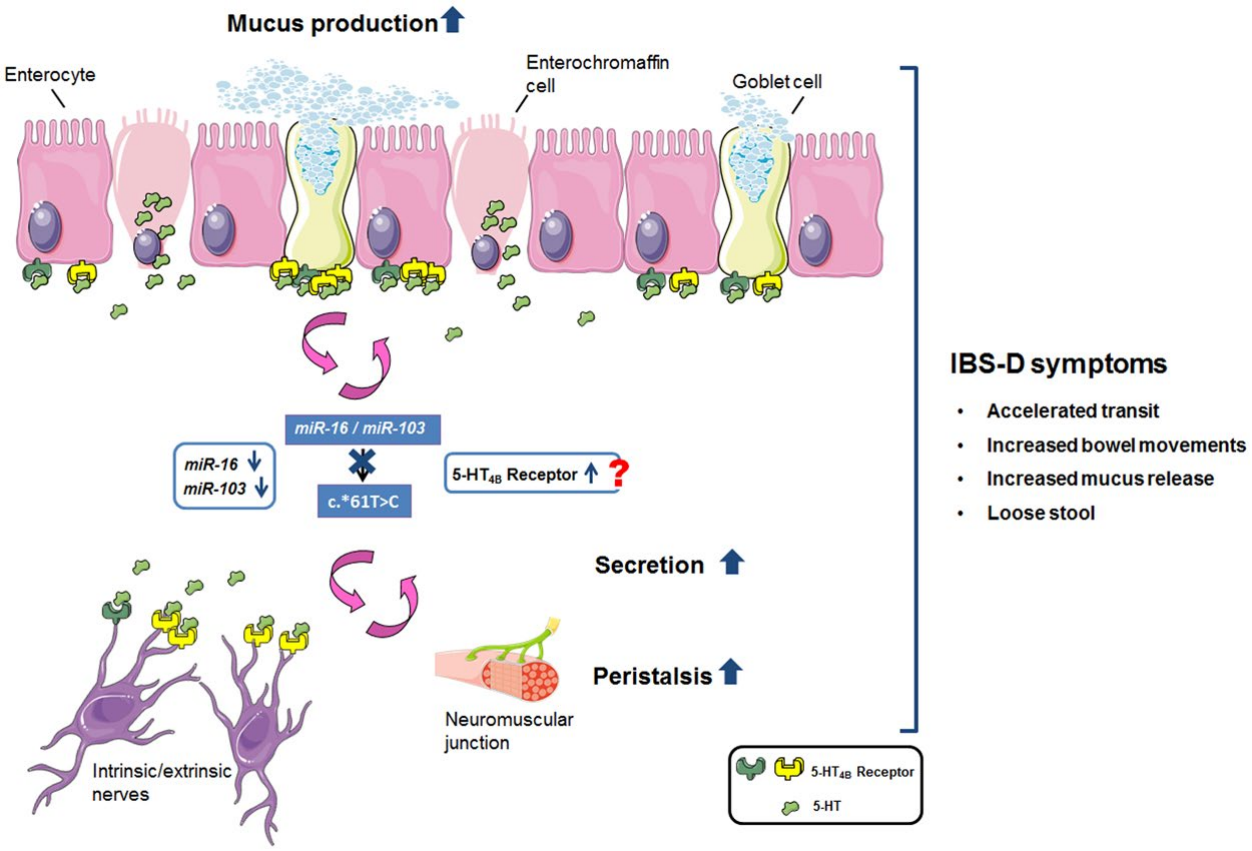
5-HT_4_ receptor mediated function in the intestine. 5-HT_4_ receptor activation promotes GI motility at different levels. Decreased miR-16 and miR-103 levels as well as hypermorphic allele variants (c.*61 T > C) may lead to elevated 5-HT_4_ receptor activity resulting in increased secretion and peristalsis in all or particular subregions and make individuals more susceptible to develop diarrhoea. Figure components were kindly provided from Servier Medical Art (http://www.servier.com). This work is licensed under the Creative Commons Attribution 3.0 Unported License. To view a copy of this license, visit http://creativecommons.org/licenses/by/3.0/ or send a letter to Creative Commons, PO Box 1866, Mountain View, CA 94042, USA. The authors acknowledge the free figure access.

Furthermore, downregulation of miR-16 and miR-103 in the jejunum of IBS-D patients adds to the relevance of this particular miRNA family in IBS and its importance in *HTR4b* regulation, presumably on a translational level since no expression changes became evident on the mRNA level. In line with this, *in vitro* results in Colo320 cells point to a secondary role of miRNA-mediated mRNA degradation concerning the mechanism of action in this regulatory process. Most importantly, the negative correlation of miR-16 and miR-103 with bowel habits and stool form, defined clinical features of IBS-D patients, fits very well with the presumed phenotypic consequences in IBS-D. More specifically, the lower the respective miRNA levels are, the higher the bowel activity and the looser the stool will be, presumably mediated via increased 5-HT_4_ receptor levels and therefore enhanced 5-HT_4_ receptor mediated signal transduction (Fig. 8).

Based on our *in vitro* data, a more pronounced effect on protein level might be anticipated contributing to the obvious clinical features. Due to the current limitations discussed above, protein expression analysis is currently impossible since no isoform-specific antibody exists (Supplementary Figure S5). As the various *HTR4* isoforms mainly differ in their respective 3′UTRs (Figure 1A)^21^, this gene seems to use these regions to regulate differential expression and receptor homeostasis. Alternative polyadenylation and varying length of 3′UTRs are involved in many cellular processes during development and differentiation^27^. The two novel isoforms provide further evidence towards a sophisticated regulatory mechanism to enable the *HTR4b_2* transcript to escape miRNA regulation. Additional 3′UTR polymorphisms may subsequently be identified, which could interfere with splicing consensus sequences or mRNA secondary structures or affect miRNA regulation^28^.

Interestingly, miR-16 has been reported to regulate other IBS-relevant genes of the serotonergic system, in particular the SERT gene *SLC6A4*, regulating serotonin bioavailability in the nervous system but also gut epithelium^29^. Also relevant due to the high comorbidity of IBS with psychiatric phenotypes, miR-16 has been reported to mediate depression and anxiety behaviours through regulation of *SLC6A4* ^29,30^. Moreover, miR-16 has lately been shown to reduce TNF-α and IL-12p40 levels, putatively suppressing mucosal inflammation and thereby resulting in the relief of symptoms in a colitis mouse model suggesting miR-16 as a potential therapeutic target for the treatment of Crohn’s disease^31^. More recently, we found impaired miR-16 expression regulation of Claudin-2 (*CLDN2*) in IBS-D, thereby contributing to disturbed gut barrier function^19^.

Putting our results into perspective, alterations in the serotonergic system in IBS patients have been described for IBS in general, as well as the different subtypes. Therefore, the *HTR4* polymorphism / miRNA level changes may also be of relevance in IBS-C patients enrolled in this study as well as other subtypes which were not included. This is based on the fact that previous studies have shown that the IBS subtype might not be stable over time since IBS symptoms, including bowel habits fluctuate. Amongst others, a one year follow up study showed that 29% of the patients switched between the IBS-D and IBS-C subtypes^32^ while another one reported that only 14% of IBS-C cases changed to IBS-D or vice versa. However, although changes between IBS subtypes are common, changes between IBS-C and IBS-D are rare^33^.One major issue in IBS are the very limited treatment options. Due to the heterogeneous origin of the disorder, therapy is mostly based on *“trial and error”* and targeting predominant symptoms rather than the whole symptom complex of IBS^12^. Consequently, molecular definition of functional variant carriers might be used for custom-tailored treatment. IBS-D patients carrying a *HTR4* SNP might benefit from 5-HT_4_ receptor-targeting compounds. According to the Medtrack database (www.medtrack.com) currently six drugs targeting 5-HT_4_Rs are explored as potential treatment for IBS. In addition, siRNA or miRNA-based drugs could be used in the future to re-adjust expression levels that have been disturbed either by decreased miRNA expression or gene variants, as seen in our study.

In conclusion, we delineate first feasible molecular mechanisms by which the miR-16 family and miR-103 as well as a non-coding, cis-regulatory variant in the *HTR4* gene may impact 5-HT_4_ receptor levels and functions, predisposing carriers to an IBS phenotype with diarrhoea symptoms. Follow-up studies will shed greater light on the role of miRNAs in functional GI disorders, the 5-HT system in general and in particular on the emerging importance of the *HTR4* isoforms.

## Material and Methods

### IBS patients and healthy controls

SNP analysis was carried out on DNAs from six case-control cohorts. Expression analyses of *HTR4b/HTR4b_2* and miRNAs in jejunal biopsies were carried out in a case-control cohort from Vall d’Hebron Institute of Research (Barcelona) (see Supplementary Table S2).

All participants were of Caucasian origin. Written informed consent was obtained from all subjects and the experiments were conformed to the principles set out in the WMA Declaration of Helsinki and the Department of Health and Human Services Belmont Report. All studies were approved by the local Ethic Committees as outlined in detail in the Supplementary Information.

### RNA isolation

Total RNA of cryoconserved or laser capture microdissected (LCM) gut resections, jejunal mucosal biopsies or cultivated cells was isolated using TRIzol Reagent (Thermo Fisher Scientific, Waltham, Massachusetts) according to the manufacturer. Total RNA samples were used for cDNA synthesis, nCounter expression analyses or 3′RACEs.

### cDNA synthesis

1 µg RNA was reverse transcribed using the SuperScript III First-Strand Synthesis System (Thermo Fisher Scientific) (ratio random hexamer/ Oligo(dT): primers 1:1). For miRNA quantification, cDNA synthesis was performed using 20 ng of total RNA with the Universal cDNA Synthesis Kit II (Exiqon, Vedbaek, Denmark).

### Polymerase chain reactions (PCRs)

Reverse transcription polymerase chain reaction (RT-PCR) and quantitative PCR (qPCR) are described in detail in the Supplementary Methods. Primer sequences are specified in Supplementary Table S1.

### Rapid amplification of cDNA ends (3′RACE)

1 µg total RNA from human ileum and colon were reverse transcribed using the ThermoScript RT-PCR System for First-Strand cDNA Synthesis (Thermo Fisher Scientific) according to the manufacturer’s instructions. 3′RACE PCRs specific for *HTR4b* were performed using the HotStarTaq DNA Polymerase protocol. First PCR was run for 15 cycles with 2 min elongation time using 1 µl of the Thermoscript cDNA product as template. 1 µl product of the first PCR run was taken as template for the following nested PCR, which was run for another 30 cycles with 2 min elongation time per cycle, respectively. Primer sequences given in Supplementary Table S1. PCR products were analysed on a 1.5% agarose gel, cloned into the pSTBlue-1 AccepTor Vector (Merck Millipore, Billerica, Massachusetts) according to the manufacturer’s instructions and sequence verified with the MegaBACE system (GE Healthcare, Little Chalfont, United Kingdom).

### Plasmid generation and mutagenesis

An existing pcDNA3.1(+) *HTR4* cDNA construct was modified in order to generate a *HTR4b* (full length) and *HTR4b_2* construct by cloning respective 3′UTRs downstream via *AfeI* (*HTR4* internal restriction site) and *NotI* (vector restriction site).

For luciferase reporter constructs, *HTR4b_2* 3′UTRs were cloned downstream of the hRluc gene in the psiCHECK-2 vector (Promega, Madison, Wisconsin) via *XhoI* and *NotI* restriction sites.

Full length *HTR4b* 3′UTRs were cloned downstream of the Rluc gene in the pRL-TK vector (Promega) via its *XbaI* restriction site. Site-specific mutagenesis was performed using the QuikChange Lightning Site-Directed Mutagenesis Kit (Stratagene, San Diego, California). All primers are listed in Supplementary Table S1.

Plasmids were purified using the PureLink HiPure Plasmid Filter Midiprep Kit (Thermo Fisher Scientific) and inserted sequences verified by Sanger sequencing using the MegaBACE system (GE Healthcare).

### nCounter expression analysis

Total RNA (up to 100 ng) of LCM colon samples and jejunal mucosal biopsies served as input material for the quantitative nCounter expression analysis (NanoString Technologies, Seattle, Washington) using a human miRNA codeset (release 1.2) and a customized codeset, as recommended by the manufacturer. The customized codeset included probes for *HTR4b, HTR4b_2* and *HTR4i* detection as well as cell type-specific markers for verification of layer specificity (Supplementary Table S4). miRNA expression data was analysed according to the manufacturer’s instructions. Normalisation was performed to the top 100 miRNA counts.

Background correction and normalisation of the customized codeset data were performed using the nSolver Analysis Software 3.0 provided by NanoString Technologies.

### Cell culture and transfection

The human cell lines HEK293T (embryonic kidney) and Colo320 (colon cancer) were maintained in Dulbecco’s modified eagle medium (DMEM, Thermo Fisher Scientific), supplemented with 10% fetal bovine serum (FBS, Thermo Fisher Scientific), 100 U/ml penicillin and 100 µg/ml streptomycin (Thermo Fisher Scientific) in a humidified atmosphere containing 5% CO_2_ at 37 °C. Transfections of HEK293 and Colo320 cells with Pre-miR miRNA precursors were performed in Opti-MEM I Reduced Serum Media (Thermo Fisher Scientific) using Lipofectamine RNAiMAX Reagent (Thermo Fisher Scientific). Co-transfection of *HTR4* constructs and Pre-miR miRNA precursors for luciferase assays and In Cell Western (ICW) experiments were carried out with polyethylenimine (PEI, Sigma-Aldrich, St. Louis, Missouri).

Transfections and harvesting of cells are described in detail in the Supplementery Methods.

The HT29-MTX-E12 cell line (kindly provided by Dr. Marguerite Clyne, University College Dublin) was cultured in DMEM, high Glucose, GlutaMAX plus 10% FCS, 1% NEAA and 1% Sodiumpyruvat (Thermo Fisher Scientific). Transfection of miRNA precursors was performed with Lipofectamine 2000 (Thermo Fisher Scientific) and CombiMag (OZBiosciences, Marseille, France) as outlined in Supplementary Methods. Pre-miR miRNA precursors are given in Supplementary Table S5.

### Luciferase assay

Luciferase assays were performed using the Dual-Luciferase Reporter Assay System (Promega) according to the manufacturer. Briefly, cells were lysed with 100 µl 1x passive lysis buffer per well and a 50 µl aliquot of each sample was measured in a Berthold Centro LB 960 luminometer. Three transfection replicates were measured per individual experiment.

### In Cell Western (ICW)

24 h after transfection cells were washed once with 1x phosphate buffered saline (PBS, Thermo Fisher Scientific) and immediately fixed with 4% paraformaldehyde (PFA, Sigma-Aldrich)/1x PBS for 15 min. Cells were washed three times and permeabilized with 0.1% Triton-X-100 (Sigma-Aldrich) in 1x PBS (5 min/wash) and subsequently blocked with Odyssey Blocking Buffer (LI-COR, Lincoln, Nebraska) for 1 h. Incubation with 1:500 diluted primary antibodies (rabbit anti-5-HT_4_ #HPA040591; mouse anti-GFP) was carried out in Odyssey Blocking Buffer for 1 h and followed by another three wash cycles with 1x PBS. Then, cells were incubated with secondary antibody solution (1:750 in Odyssey Blocking Buffer; donkey anti-rabbit IRDye 800CW and donkey anti-mouse IRDye 680CW, LI-COR) for 1 h, protected from light. Cells were washed three times with 1x PBS, scanned with a LI-COR Odyssey Infrared Imaging System and analysed by the software provided by the manufacturer. An antibody list is given in the Supplementary Table S6.

### Tissue material

Unaffected, normal tissue from stomach, duodenum, jejunum, ileum and colon was obtained from three female and two male patients (55-77 years old, two colon adenocarcinomas, one small intestine adenocarcinoma, one gastric carcinoma and one pancreatic adenocarcinoma; tissue was obtained from the GEZEH tissue bank (www.gezeh.de) and approved by the local Ethic Committee) and used for RNA extraction, subsequent RT-PCR analysis and 3′RACEs.

Four unaffected colon samples (two male and two female patients, 56–70 years old; two sigma diverticulitis, one rectum carcinoma and one hemicolectomy) were used for laser captured microdissection and subsequent nCounter expression analysis.

Jejunal mucosa samples from IBS-D patients and healthy controls were obtained as described earlier^19,34^.

### Laser captured microdissection (LCM) and pressure catapulting

Fresh frozen colon samples were cut into 18 µm thick sections using a cryostat (Leica CM1850, Leica Microsystems, Wetzlar, Germany) and processed as following: the sections were mounted on membrane slides (PEN-membrane, 1 mm glass, Carl Zeiss MicroImaging GmbH) and incubated for 10 min at −20 °C in RNAlater-ICE (Ambion, Thermo Fisher Scientific). For further preservation, samples were fixed in ethanol and stained in cresyl violet acetate (1% (w/v) in ACS-grade ethanol (all from Sigma-Aldrich) for 15 s. Subsequently, the slides were washed in ethanol and incubated for 5 min in xylene (Carl Roth, Karlsruhe, Germany). After air-drying, the slides were mounted on the stage of an inverse microscope which is a component of a Microbeam LMPC System (Carl Zeiss MicroImaging GmbH, Oberkochen, Germany). We employed the RoboLPC method to microdissect and capture the appropriate tissue fragments (approx. 10 mm^2^ epithelium or *lamina propria* cells, ~100,000–250,000 cells; approx. 15 mm^2^ *myenteric plexus* or muscular cell layer, ~100,000–250,000 cells).

### Statistics

#### Statistical analysis of genotyping data

Comparison of genotype frequencies, association analyses and tests for deviation from the Hardy-Weinberg Equilibrium (HWE) were performed as described previously^15^.

#### Statistical analysis for luciferase, qPCR, ICW and goblet cell data

Two-tailed parametric tests were used as appropriate (unpaired *t*-test, one-way ANOVA followed by Bonferroni correction post-hoc test) using GraphPad Prism 5.0 software (GraphPad Software, Inc., La Jolla; California). Besides, a Mann-Whitney *U* test and an unpaired t-test with Welch’s correction were applied as indicated in the figure legends.

Relationships between clinical features (bowel movement, stool form) and miRNA expression were assessed by Spearman’s correlation rho. Data are expressed as mean ± standard error of the mean (SEM), unless stated otherwise; p-values of <0.05 (*p < 0.05) were considered statistically significant.

### In silico analysis of miRNA binding sites

Comparative *in silico* analyses of miRNA binding sites in the *HTR4* gene were performed using the online prediction tools miRWalk^35,36^, RegRNA 1.0^37^, miRanda^38^ and TargetScan4.2 (www.targetscan.org). Putative miRNA binding sites predicted by at least two different algorithms were taken into account.

A summary of all samples and applied experiments is given in the Supplementary (Supplementary Figure S6).

The datasets generated during and/or analysed during the current study are available from the corresponding author on reasonable request.

## Electronic supplementary material

Supplementary information
