# Supplementary material for: miR-16 and miR-103 impact 5-HT4 receptor signalling and correlate with symptom profile in irritable bowel syndrome
Source: Sci Rep. 2017 Oct 31;7:14680. doi: 10.1038/s41598-017-13982-0 (PMC5665867; doi:10.1038/s41598-017-13982-0)
Supplement: Supplementary file 1 — Supplementary information [file 41598_2017_13982_MOESM1_ESM.pdf]

Supplementary Information

miR-16 and miR-103 impact 5-HT_4 receptor signalling and correlate with symptom profile in irritable bowel syndrome

Carolin Wohlfarth[1,*,§], Stefanie Schmitteckert[1,*,§], Janina D. Härtle[1], Lesley A. Houghton[2,3,§], Harsh Dweep[4,5], Marina Fortea[6], Ghazaleh Assadi[7], Alexander Braun[1,§], Tanja Mederer[1,§], Sarina Pöhner[1], Philip P. Becker[1], Christine Fischer[8], Martin Granzow[8], Hubert Mönnikes[9,§], Emeran A. Mayer[10,§], Gregory Sayuk[11], Guy Boeckxstaens[12,§], Mira M. Wouters[12,§], Magnus Simrén[13,§], Greger Lindberg[14,§], Bodil Ohlsson[15], Peter Thelin Schmidt[16], Aldona Dlugosz[14], Lars Agreus[17], Anna Andreasson[18,19], Mauro D'Amato[20,21,§], Barbara Burwinkel[22,23], Justo Lorenzo Bermejo[24], Ralph Röth[1,25], Felix Lasitschka[26], Maria Vicario[6,§], Marco Metzger[27,28], Javier Santos[6,§], Gudrun A. Rappold[1], Cristina Martinez[1,6,*,§] and Beate Niesler[1,25,*,§]

Table of contents:

1. Materials
1.1 Supplement supplementary tables
2. Methods
3. Results
4. Supplement supplementary Figures

1. Materials

1.1 Supplement supplementary tables

Supplement Table S1. Primer sequences

Amplicon	Primer name	Sequence 5' > 3'
HTR4a	HTR4aEx6for	AGCAGGCCTCAGTCGGCAGA
	HTR4aEx11rev	GCCAGGGTGACCTGTTCATGCA
HTR4b	HTR4b_ex6for	CTGGGCCAGACTGTCCCTTG
	HTR4b_ex8rev	AAGACAGGAGCGACGCCTCTG
HTR4d	HTR4dEx6for	GAGAGCAGGCCTCAGTCGGC
	HTR4dEx9rev	TTGGCCACAGTCCTCAAGGAGCTC
HTR4g	HTR4gEx10for	GGCTGTTCCCCTGTCTCCAGCTTC
	HTR4gEx11rev	CTCCTCTGGGCTCTCAGCCCCTA
HTR4i	HTR4iEx7for	GCCAGAGCGGGTCCATTCTCAGG
	HTR4iEx8rev	AAGCAAGACAGGAGCGACGCC
HTR4b_2	HTR4b_1309_for	GCTCAGCCCAGTGACACTTA
	HTR4b_1422_splice_rev	TTCCAGAACGGGTGCAGTC
HTR4b_3	HTR4b_2185_splice_for	GGCTAGCAACATCTTCTGGAAC
	HTR4b_2973_rev	GGCATTTGGATGGTTTGGTCA

HTR4b_2 full	HTR4bEx2_for	CCCTGTGGGCATTGAAATCCAAC
	HTR4b_Ex8_2912_rev	AGTTCCAGAACTAAAGTAAACAACA
ARF1	ARF1for	GCCAGTGTCCTTCCACCTGTC
	ARF1rev	GCCTCGTTCACACGCTCTCTG
HTR4b (qPCR)	HTR4b_qPCR_Ex6_for	TGAGCGCTACCGAAGACCTTCCA
	HTR4b_qPCR_Ex8_rev	TGCTGGCGGGTGACACTGAC
HPRT1 (qPCR)	HPRT1_for	TGATAGATCCATTCCTATGACTGTAGA
	HPRT1_rev	AAGACATTCTTTCCAGTTAAAGTTGAG
SDHA (qPCR)	SDHA_for	TGGGAACAAGAGGGCATCTG
	SDHA_rev	CCACCACTGCATCAAATTCATG
RACE Primer (Oligo(dT) + Adapter)	dT-Ada_wobble	GGCCACGCGTCGACTAGTACTTTTTTT TTTTTTTTTTTTTTTTTTTTTTT VN
RACE Primer (Adapter)	AUAP	GGCCACGCGTCGACTAGTAC
HTR4b_GSP_RACE_1	HTR4b_1344_3RACE_for	GACCCAGAAGACAGCCATGC
HTR4b_GSP_RACE_2 nested	HTR4b_1355_3RACE_for	CAGCCATGCCTCCGAAAGA
c.*61C	HTR4_61C_mut_for	GCCAGGTCCTAAGCCGCTGCTTGTGCGCGACTG
	HTR4_61C_mut_rev	CAGTCGCGCACAAGCAGCGGCTTAGGACCTGGC
mut1	HTR4_61_mut_complete_for	GGCCAGGTCCTAAGCTGCTCGGTGTGCGCGACTGC
	HTR4_61_mut_complete_rev	GCAGTCGCGCACACCGAGCAGCTTAGGACCTGGCC
mut4	HTR4b_2436_complete_mut_for	TGCCGCCCTGGCTGTGCGCTAAACAAGCTGTGAGATCT
	HTR4b_2436_complete_mut_rev	AGATCTCACAGCTTGTTTAGCGCACAGCCAGGGCGGCA
mut2	HTR4_2561_complete_mut_for	CTGTTTCTAAGACAACAGTATGCTCGGATAGCCGTCTGCTACCTCG
	HTR4_2561_complete_mut_rev	CGAGGTAGCAGACGGCTATCCGAGCATACTGTTGTCTTAGAAACAG
HTR4b_full_length 3'UTR/pRL-TK	HTR4b_3UTR_XbaI_for	ATTATCTAGAGCCCCTGGGACAATGACCCAG
	HTR4b_Ex8_2928_XbaI_rev	ATTATCTAGATCTTCCTGTCGGTTTCAGTTCCAGA
HTR4b_2 3'UTR/psiCHECK2	HTR4b_3UTR_XhoI_for	ATTACTCGAGGCCCCTGGGACAATGACCCAG
	HTR4b_2973_NotI_rev	ATTAGCGGCCGCGGCATTTGGATGGTTTGGTCA
HTR4b_full_length 3'UTR/pcDNA3	HTR4b_Ex6_ClaI_for	TGCTGTGATGATGAGCGCTACCG
	HTR4b_Ex8_NotI_rev	ATTAGCGGCCGCTCTTCCTGTCGGTTTCAGTTCCAGA
HTR4b_2 3'UTR/pcDNA3	HTR4b_Ex6_ClaI_for	TGCTGTGATGATGAGCGCTACCG
	HTR4b_2973_NotI_rev:	ATTAGCGGCCGCGGCATTTGGATGGTTTGGTCA

Supplement Table S2. Tested IBS cohorts

Cohort	Genotype	female	male	Age yrs (mean)	Rome II/III
UK	IBS-D	67	31	18-66 (41.6)	II
	IBS-C	95	5	18-65 (40.1)	
	controls	60	32	18-63 (35.6)	
Germany	IBS-D	72	42	19-79 (44.7)	III
	IBS-C	40	8	16-71 (45.5)	
	controls	435	408	25-70 (34.1)	
USA (EAM)	IBS-D	28	8	20-64 (39.6)	II/III
	IBS-C	29	3	20-58 (39.8)	
	controls	60	38	19-58 (31.1)	
Belgium	IBS-D	323	121	18-70 (44.3)	II
	IBS-C	305	25	18-81 (43.7)	
	controls	506	158	18-83 (41.1)	
USA (GS)	IBS-D	27	14	20-83 (49.9)	III
	IBS-C	15	5	28-64 (45.9)	
	controls	48	46	18-81 (55.9)	
Sweden	IBS-D	67	32	20-72 (40.6)	II/III
	IBS-C	75	9	20-78 (43.7)	
	controls	164	286	19-71 (45.3)	
Barcelona	IBS-D	19	14	22-51 (35.5)	III
	controls	15	11	19-53 (30.6)	

IBS, Irritable bowel syndrome; D, diarrhoea; C, constipation; yrs, years; EAM, Emeran Anton Mayer; GS, Gregory Sayuk.

Supplement Table S3. Sequencing primers for mutational analysis

Target Region 3‘UTR	Primer name	Sequence (5‘ > 3‘)	3‘UTR/ Amplicon (in bp)	T_A (°C)
HTR4 a	HTR4 a UTR in for<br>HTR4 a UTR in rev	TGC AAA GAA AGG AAG TCA AGA<br>CCT CCA TGT ACC TCC TCT GG	153/330	57,5
HTR4 b&i	HTR4 b UTR in for<br>HTR4 b UTR in rev	ATG GCA CAG AGT GTT CCT GC<br>TCT GGG AAG AGG GAG TGT TG	242/433	59

Supplement Table S4. Sequences of nCounter probes

Gene	Accession No.	Probe
HTR4b	NM_000870.5	CATTAATGGATCCACACATGTACTAAGGGATGCAGTG GAGTGTGGTGGCCAGTGGGAGAGTCAGTGTCACCCG CCAGCAACTTCTCCTTTGGTGGCTGCT
HTR4b_2	n.a.	GCCTCCGAAAGAGGGCCAGGTCCTAAGCTGCTGCTT GTGCGCGACTGCACCCGTTCTGGAACTGAAACCGAC AGGAAGAAACTTTGGAATAAGGAAGAGA
HTR4i	NM_001040173.2	TGGATCCACACATGTACTAAGGACAGATTTTCTGTTT GACAGAGACATTTTAGCAAGGTATTGGACAAAGCCTG CCAGAGCGGGTCCATTCTCAGGCACA
ARF1	NM_001024227.1	CAATTCTGCATGGTCACAGTAGAGATCCCCGCAACTC GCTTGTCCTTGGGTCACCCTGCATTCCATAGCCATGT GCTTGTCCCTGTGCTCCCACGGTTCC

SDHA	NM_004168.2	CAGTGGCCAGGGAGCGTGGCACTTACCTTTGTCCCT TGCTTCATTCTTGTGAGATGATAAAACTGGGCACAGC TCTTAAATAAAATATAAATGAACAAAC
TBP	NM_003194.4	CAGCTTCGGAGAGTTCTGGGATTGTACCGCAGCTGC AAAATATTGTATCCACAGTGAATCTTGGTTGTAAACTT GACCTAAAGACCATTGCACTTCGTGC
SNX17	NM_014748.2	CTTTCCTTGTCCCCTGGGCTGGCTGCACAGAGGATT GCCCCTTCTCTTTTCAGAGCTGGCCCTCGATGCCAAA TTAGCATTTAGTATTTTGCACAAAGTC
PGK1	NM_000291.2	GCAAGAAGTATGCTGAGGCTGTCACTCGGGCTAAGC AGATTGTGTGGAATGGTCCTGTGGGGGTATTTGAATG GGAAGCTTTTGCCCGGGGAACCAAAGC
UBB	NM_018955.2	TTTGCGCTCCGCCAGCCCGGAGCATTTAGGGGCGGT TGGCTTTGTTGGGTGAGCTTGTTTGTGTCCCTGTGGG TGGACGTGGTTGGTGATTGGCAGGATC

Supplement Table S5. miRNA molecules

Type	miRNA	Accession No.
Pre-miR miRNA Precursor	hsa-miR-15b	MIMAT0000417
	hsa-miR-16	MIMAT0000785
	hsa-miR-497	MIMAT0002820
	hsa-miR-103	MIMAT0000101
Pre-miR miRNA Precursor	Negative Control #1	Cat. # AM17110
Pre-miR customized miRNA	miR16 A>G	n.a.

Supplement Table S6. Antibodies

Target	Company	Name/Number #	raised in	Type/labelled with
5-HT_4 receptor	MoBiTec	LS-A655	rabbit	polyclonal
5-HT_4 receptor	Abcam	ab87337	rabbit	polyclonal
5-HT_4 receptor	Abcam	ab60359	rabbit	polyclonal
5-HT_4 receptor	Abcam	ab101910	rabbit	polyclonal
5-HT_4 receptor	Sigma-Aldrich	HPA040591	rabbit	polyclonal
5-HT_4 receptor	Sigma-Aldrich	SAB4501480	rabbit	polyclonal
Myc-Tag	Cell Signalling	2276	mouse	monoclonal
ß-Tubulin	Sigma-Aldrich	T-4026	mouse	monoclonal

GFP	Sigma-Aldrich	G 6539	mouse	monoclonal
mouse IgG	LI-COR	926-32222<br>IRDye 680	donkey	monoclonal/<br>IRDye680
rabbit IgG	LI-COR	926-32213<br>IRDye 800CW	donkey	polyclonal/<br>IRDye800CW
mouse IgG	Thermo Fisher	A11029, Alexa Fluor 488	goat	monoclonal/<br>Alexa Fluor 488
rabbit IgG	Thermo Fisher	A11036, Alexa Fluor 568	goat	polyclonal/<br>Alexa Fluor 568

2. Methods

IBS patients and healthy controls

Informed consent was obtained from all participants and local ethics committees approved the study protocol as follows: UK, Manchester: NHS National Research Ethics Service, South Manchester Research Ethics Committee, Genetics of Functional GI Disorders, 09/H1003/1; Germany, Heidelberg: Ethical Committee, Medical Faculty of the University Hospital Heidelberg, S067/2010; USA, St-Louis, WA: Washington University St.Louis, Human Research Protection Office, IRB ID #: 201103220; Ethics Committee of Hospital Universitari Vall d´Hebron (PR(AG)159/2011), Karolinska Institutet's Ethics Review Board, dnr 2009/1059-31/3, USA Los Angeles, CA, University of California Los Angeles, HORPP Office of the human research protection program; IRB#12-001802-CR-00004; Furthermore, a cohort of IBS patients and controls from UK/US/Canada were included in this study. All samples were kindly provided by Glaxo Smith Kline (UK, GenIBS/2005/0000/01).

Preparation of genomic DNA

Genomic DNA was prepared from blood or saliva samples taken from both patients and healthy controls using standard protocols as previously described (Kapeller et al, 2008; Kilpatrick et al, 2011).

Sequencing of *HTR4* isoforms

Polymerase Chain Reaction (PCR)

PCRs were performed in 12.5 µl volumes containing 25-50 ng of genomic DNA as template, 6.25 pmol of each primer, 200 µM dNTPs (MBI Fermentas, St. Leon-Rot, Germany), HotStarTaq PCR Buffer 10x concentrated (contains Tris-HCl, KCl, $(NH_4)_2SO_4$, 15 mM $MgCl_2$), and 0.625 U of HotStarTaq DNA Polymerase (Qiagen, Venlo, Netherlands). Thermal cycling was performed in a Mastercycler gradient thermal cycler (Eppendorf, Hamburg, Germany). Annealing temperatures (T_A) and sequences of the 3'UTR specific *HTR4* primers are shown in Supplement Table S3. Cycling conditions were: Initial denaturation at 95°C for 15 min followed by 35 cycles of 94°C for 30 s, T_A for 30 s and 72°C for 30 s. The final extension step was at 72°C for 10 min. A 3 µl aliquot of each PCR product was analysed on a 1.5 % agarose gel and imaged using the Quantum-1100 Imaging System and the Quantum Capt software (VWR, Darmstadt, Germany).

Purification and direct sequencing of PCR products

A 2.5 µl aliquot of PCR product was treated with 0.5 U FastAP Thermosensitive Alkaline Phosphatase (Fermentas) and 5 U exonuclease I (ExoI; MBI Fermentas) for 15 min at 37°C followed by inactivation at 80°C for 15 min. 2 µl of the ExoI/SAP-treated PCR product was used for direct sequencing using the DYEnamic ET Terminator Cycle Sequencing Kit

according to the manufacturer's protocol (GE Healthcare, Little Chalfont, United Kingdom). The MegaBACE 1000 sequencer and the software provided by the manufacturer (GE Healthcare) were used for analysis of the sequence reaction products.

KASP genotyping assay

SNP genotyping was performed with the KASP assay system (KBiosciences, Ltd, Hoddesdon, United Kingdom) as recommended by the manufacturer using customized assays. Thermal cycling was carried out in Mastercycler *vapo.protect* thermal cyclers (Eppendorf). An initial 15 min incubation at 94°C was followed by 20 cycles consisting of 10 s at 94°C, 5 s at 57°C, and 10 s at 72°C and a following 28 cycles consisting of 10 s at 94°C, 20 s at 57°C, and 40 s at 72°C. After thermal cycling, results were analysed using the fluorescence plate reader of the 7500 Fast Real-Time PCR System (Applied Biosystems, Foster City, California). 10 % of the samples were repeated for quality control constraints and could be confirmed.

Reverse transcription polymerase chain reaction (RT-PCR)

RT-PCRs were performed in 25 µl volumes with 1 µl cDNA (1:3 dilution). Final concentrations per reaction comprised 0.4 µM primer mix and 250 µM dNTPs in 1x HotStarTaq PCR buffer and 1.25 U HotStarTaq (Qiagen, Venlo, Netherlands). Thermal cycling was carried out using Mastercycler *vapo.protect* (Eppendorf, Hamburg, Germany). Cycling conditions were: Initial activation step at 95°C for 15 min followed by 35 cycles (for sequencing) or 40 cycles (RT-PCR) of 30 s 94°C, 30 s 60°C and 30 s 72°C and a final elongation of 10 min at 72°C. A 5 µl aliquot of each PCR product was analysed on a 1.5 % agarose gel and imaged using the Quantum-1100 Imaging System and the Quantum Capt software (VWR, Darmstadt, Germany). Oligonucleotide sequences are given in Supplementary Table S1.

Quantitative PCR (qPCR)

qPCR was performed in triplicates (technical replicates) on a 7500 Fast Real-Time PCR System (Applied Biosystems, Foster City, California) using SYBR Green ROX dye (Thermo Fisher Scientific) according to manufacturer's instructions. All values were normalised to *SDHA* (NM_004168.2; gene coding for succinate dehydrogenase complex, subunit A, flavoprotein*)* or 18s RNA. Primer sequences are specified in Supplementary Table S1.

qPCR was also applied to assess miRNA expression in samples from 14 IBS-D *vs.* 18 healthy controls. It was performed on an ABI PRISM 7500 FAST Sequence Detection System (Thermo Fisher Scientific) using LNA-primers (Exiqon). Each sample, including distilled water as negative control, was run in triplicates and data was analysed by the $2^{-\Delta\Delta Ct}$

method. The expression of each miRNA was normalised to the average of three selected reference small RNAs (U6, RNU5G and SNORD44) and the fold-change was calculated individually with respect to the average of the healthy control group.

Purification and direct sequencing of PCR products

Samples positive for the *HTR4b/i* c.*61C allele were validated by Sanger sequencing as follows: A 2.5 µl aliquot of PCR product was treated with 5 U Exonuclease I (Fermentas, St-Leon-Rot, Germany) and 0.5 U Thermosensitive Alkaline Phosphatase (FastAP, Fermentas) in a final volume of 4 µl for 15 min at 37°C followed by inactivation at 85°C for 15 min. 2 µl of the respective ExoI/FastAP reaction were used for direct sequencing using the DYE-namic ET Terminator Cycle Sequencing Kit according to the manufacturer's protocol (GE Healthcare, Little Chalfont, United Kingdom). For sequence analysis the MegaBACE 1000 sequencer (GE Healthcare) and Geneious software (version 5.3.6 created by Biomatters, available from http://www.geneious.com) were used.

Transfections

To investigate the effect of the different miRNAs on *HTR4b* transcript levels, Colo320 cells were seeded into 6-well plates with approximately 30 % cell density in Opti-MEM I Reduced Serum Media (Thermo Fisher Scientific) + 10 % FBS one day prior to transfection. Cells were transfected with Ambion Pre-miR miRNA precursors (hsa-miR-15b-5p (MIMAT0000417), hsa-miR-16 (MIMAT0000785), hsa-miR-497-5p (MIMAT0002820), hsa-miR-103a-3p (MIMAT0000101), negative control miR #1, see Supplementary Table S5, all from Thermo Fisher Scientific) at 10 nM final concentrations using Lipofectamine RNAiMAX Reagent (Thermo Fisher Scientific). Cells were harvested 72 h after transfection in TRIzol and subsequently processed for RNA isolation.

Luciferase assays with *HTR4b* full length 3'UTR reporters were carried out using pRL-TK-*HTR4b* 3'UTR and pGL3-control vectors. Cells were seeded at approximately 30 % density one day prior to transfection into 24-well plates. Per well, 400 ng of the respective pRL-TK *HTR4b* 3'UTR construct and 100 ng of the reference construct pGL3-control were co-transfected using 4 µg of polyethylenimine (PEI, Sigma-Aldrich, St. Louis, Missouri) per 1 µg of DNA along with Ambion Pre-miR miRNA precursors (10 nM final concentration). Luciferase assays with short *HTR4b*_2 3'UTR reporters were carried out using a psiCHECK-2-*HTR4b_2* 3'UTR vector (Promega) as follows: cells were seeded at approximately 30 % density one day prior transfection into 96-well plates. Per well, 25 ng of the respective psiCHECK-2 vector were co-transfected using 4 µg of PEI (Sigma-Aldrich) per 1 µg of DNA along with Ambion Pre-miR miRNA precursors (10 nM/40 nM final concentration). Cells were harvested for luciferase read-out 48 h after transfection.

In Cell Western (ICW) experiments were carried out using HEK293T cells seeded at 80 % density onto 0.01 % Poly-L-Lysine (Sigma-Aldrich)-coated 96-well plates one day prior to transfection. Per well 100 ng of pcDNA3.1(+) 5-HT_4 receptor expression vector were co-transfected with 50 ng of pEGFP-C1 (Clontech Laboratories, Mountain View, California; used as transfection control) using PEI as described before. ICW was carried out 24 h after transfection.

The HT29-MTX-E12 cell line (kindly provided by Dr. Marguerite Clyne, University College Dublin) was cultured in DMEM, high Glucose, GlutaMAX plus 10 % FCS, 1 % NEAA and 1 % Sodiumpyruvat (Thermo Fisher Scientific). Prior transfection, 150.000 cells were seeded in a 6-well plate with 1.8 ml complete growth medium. 2 µl Lipofectamin 2000 (Thermo Fisher Scientific) and 25 nM miRNA (hsa-miR-16 (MIMAT0000785), miR-16 A>G, hsa-miR-103a-3p (MIMAT0000101), negative control miR #1, see Supplementary Table S5) were diluted in 100 µl Opti-MEM medium (Thermo Fisher Scientific), respectively. Both mixes were pooled, added to 1 µl CombiMag (OZBiosciences, Marseille, France) and mixed by vigorous

pipetting. After 30 min incubation at RT, the complex was added to the cells. The cell culture plate was placed upon the magnetic plate and incubated at 37°C for 20 min in the incubator. After 2 days, cells were washed twice in cold PBS, harvested and stored in 100 µl TRIzol at -80°C until further use.

Immunofluorescence analysis

Cells were seeded on coverslips coated with poly-L-lysine (Thermo Fisher Scientific) at approximately 30 % density in 12-well plates. If necessary, cells were transiently transfected, as earlier described, the day after seeding and used the following day. Immunofluorescence staining was performed at RT according to the following protocol: Briefly, cells were washed once with 1x PBS, fixed with 4 % paraformaldehyde (PFA) for 15 min, subsequently, cells were washed twice with 30 mM Glycin/1x PBS pH 7.5. After that, cells were permeabelised with 1x PBS/0.1 % Triton X-100 for 5 min, followed by one washing step with 1x PBS. After that, the primary antibody (1:100 in 1x PBS, Supplement Table S6) was applied overnight. Next day, the cells were washed three times with 1x PBS and incubated with the respective secondary antibody (1:500 in 1x PBS, Supplement Table S6) for 1 h. Then, cells were washed three times with 1x PBS and a nucleic counterstain was performed with Hoechst 33342 for 3 min. Finally, cells were washed twice with 1x PBS, air-dried and mounted using Mowiol (Calbiochem; Merck, Darmstadt, Germany).
Fluorescence microscopy was performed using a Nikon Eclipse 90i upright automated microscope with DIC and epi-fluorescence optics at the Nikon Imaging Centre of the Heidelberg University. Subsequent analysis was performed using the NIS-Elements Advanced Research software (Nikon, Minato, Tokio). Further image processing was carried out using ImageJ 1.43.

Western blot

Cells were washed once with 1x PBS, harvested using a cell scraper, centrifuged (13.000 rpm, 3 min) and the pellet lysed in 400 µl cell lysis buffer on ice for 30 min. The lysate was centrifuged at 13.000 rpm for 1 min and the supernatant transferred to a new tube for further processing. To determine the protein concentration of the cell lysates, a BCA-assay (BCA Protein Assay Kit, Pierce) was carried out. Protein lysates (each 11.5 µg) were separated on the NuPAGE system (Thermo Fisher Scientific, Bis-Tris Mini gels) as recommended by the manufacturer. Afterwards, the protein was transferred onto a PVDF membrane (Immobilon-FL Membrane, Merck Millipore, Billerica, Massachusetts) and fluorescence detection was carried out using the Odyssey Infrared Imaging System of LI-COR (Lincoln, Nebraska).

3. Results

Mutational analysis *HTR4* 3'UTRs

Sequencing of the discovery sample from the UK (Supplement Table S1) included DNA samples of 98 IBS-D; 100 IBS-C and 91 control samples. The GI-relevant isoforms *HTR4a, b,* and *i* were analysed. For this purpose, we restricted our analysis to the stop coding near portions of the 3'UTRs (primers given in Supplement Table S3) and revealed the tested regions to be highly conserved in all isoforms. Only the SNP c.*61T>C (rs201253747) affecting the isoforms *HTR4b* and *HTR4i* was exclusively found in IBS-D patients. None of the other clinically defined individuals, neither controls nor IBS-C patients, carried this variant.

5-HT4 receptor antibody characterisation

Western blot analysis

As on protein level, only *in vitro* assays (In Cell Western, luciferase assays) have been used to analyse the regulation of *HTR4b* by miR-16 and miR-103, we aimed at confirming differential expression regulation by the miR-16 family by Western blot (WB) analysis of *HTR4* endogenously expressing cell lines and patient material. Thus, WB analysis was firstly applied to confirm the regulation of endogenous 5-HT_4 receptor levels in miR-16 transfected Colo320 cells. Since to date, no convincing examples for 5-HT_4 receptor WB detection had been reported, we aimed to establish a suitable protocol by verifying the receptor expression in pcDNA3 myc-HTR4b transfected HEK293T cells via the myc-tag. Subsequent probing of respective blots with different anti-5-HT_4 receptor antibodies only identified one single antibody (ab101910, Abcam, Cambridge; United Kingdom) to specifically detect 5-HT_4 receptors. However, the specific 5-HT_4 receptor signal at approximately 45 kDa size was accompanied by multiple strong bands that were detectable in both protein lysates of transfected and untransfected cells indicating non-specific cross-reactivity (Supplementary Figure S2A). For further examination, protein lysates from Colo320 cells were blotted onto a PVDF membrane and only probed with the ab101910 antibody. Here too, additional bands appeared apart from the 5-HT4 receptor signal at 45 kDa, which could either represent other 5-HT4 receptor isoforms, receptor-dimers, differentially modified receptors (such as glycosylated, palmitoylated, or phosphorylated receptors) or unspecific cross-reactions of the antibody (Supplementary Figure S2B). Due to the high signal heterogeneity obtained in these experiments - either through unspecific signals or receptor modifications - the WB method was not considered to be suitable to quantify changes of 5-HT_4 receptor levels upon miRNA transfections and miRNA downregulation.

Does a 5-HT_{4b} receptor specific antibody exist?

As outlined earlier, on protein level, most of the 5-HT_4 receptor isoforms vary only at their very C-terminal end (Figure 1A'). Until recently, all commercially available antibodies exclusively targeted the "common" region, which is identical in all isoforms. Consequently, expression data distinguishing between isoforms has so far been restricted to RNA-based methods and has been unable to reveal the actual picture on receptor protein level.

Since lately, Sigma-Aldrich has offered a novel anti-5-HT_4 receptor antibody (HPA040591, Sigma-Aldrich, St. Louis; Missouri) raised in rabbit with an immunogene epitope representing the very C-terminal amino acid sequence of the 5-HT4b isoform (aa 319-387), including 39 "common" and 30 "b-specific" amino acids. To test whether this antibody solely detects 5-HT4b receptors, we co-stained transiently transfected U2OS cells (either with a pRK5 myc-*HTR4a* construct (kindly provided by Prof. Joël Bockaert, Montpellier, France) or a pcDNA3 myc-HTR4b construct) with the Sigma-Aldrich HPA040591 antibody and a c-Myc-Tag antibody (clone 9B11, Cell Signaling Technology, Danvers, Massachusetts). Both 5-HT_{4a} and 5-HT_{4b} receptor expressing cells were stained by the Sigma-Aldrich antibody as shown in Supplementary Figure S3 by the yellow myc-/5-HT_4 receptor co-staining. Hence, the described antibody proved not to be suitable to distinguish between different receptor isoforms in further immunofluorescence experiments.

Acknowledgements

We acknowledge the support of Dr. Theadore Ptak, Renee Henry, Ellen Goldstein, Cindy Lee, Deborah Roach, Jacqueline Rabuzin and Elizabeth Crosland (Toronto Digestive Disease Associates (TDDA Inc.), Toronto, Ontario, Canada), Dr. Mark Silverberg and Lori Baladjay (Mount Sinai Hospital, Toronto, Ontario, Canada), Dr. Yehuda Ringel, Dr. Robert Sandler, Alesia N. Aileo, Sarah Causey and Sarah Yeskel (University of North Carolina at Chapel Hill, NC, USA) as well as the staff at Ersta Hospital (Stockholm, Sweden) for collecting samples and acquiring data and Dr. Rachel Gibson and Dr. George Dukes (GlaxoSmithKline) for logistic support as well as for patient recruitment and clinical data collection. Besides, we kindly acknowledge Georg Stöcklin and Sven Diederichs as thesis advisory committee members in the *'The Hartmut Hoffmann-Berling International Graduate School of Molecular and Cellular Biology graduate school'* for helpful discussions. We also thank Jutta Scheuerer, Verena Wahl and Sabine Wilhelm for expert technical assistance. FL was funded by DFG/SFB 938 TP Z2 (www.gezeh.de). Thanks to Milagros Gallart, Adoración Nieto, Laura Hernández and Sara Méndez from Hospital Universitari Vall d'Hebron for their invaluable assistance in the performance of jejunal biopsies. GB is supported by a grant from the Flanders Research Foundation (FWO, Odysseus). MW is supported by a FWO postdoctoral research fellowship. MD is funded by the Swedish Research Council (VR). MS

is supported by the Swedish Medical Research Council (grants 13409, 21691 and 21692), the Marianne and Marcus Wallenberg Foundation, and the University of Gothenburg, Centre for Person-Centred Care (GPCC), Sahlgrenska Academy, University of Gothenburg and by the Faculty of Medicine, University of Gothenburg; MF is supported by Vall d'Hebron Institute of Research (Pred-VHIR-2014-018); MV and JS are supported by Fondo de Investigación Sanitaria and CIBERehd, Instituto de Salud Carlos III, Subdirección General de Investigación Sanitaria, Ministerio de Economía y Competitividad (PI13/00935, PI14/00994); CM is supported by Ministerio de Economía y Competitividad, subprograma estatal de incorporación (IJCI-2015-26099).

4. Supplementary Figures

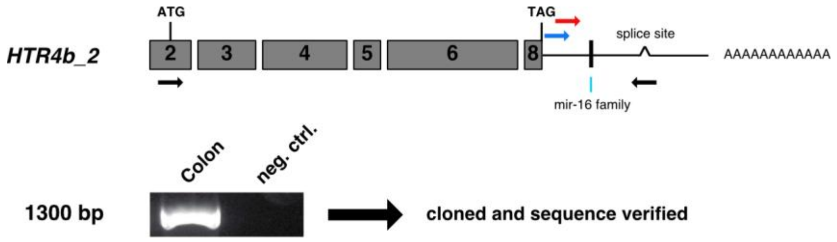

Supplementary Figure S1. Amplification of exon 2 - 8 of the *HTR4b_2* transcript. RT-PCR was performed using a forward primer residing at the start codon ATG and a *HTR4b_2* specific reverse primer (black arrows depict primer positions). The amplification product was purified and cloned into a pSTBlue-1 vector for subsequent sequencing. Blue and red arrows indicate primers used for 3'RACE.

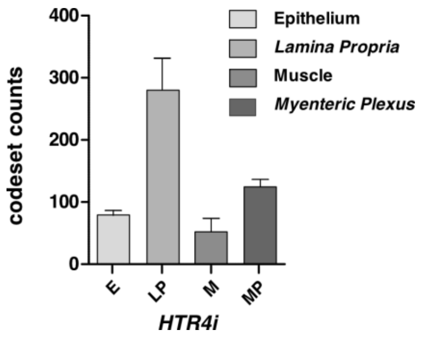

Supplementary Figure S2. nCounter expression analysis of *HTR4i* in unaffected LCM human colonic subregions. Values are means ± SEM of nCounter derived codeset counts from total RNA of unaffected tissue obtained from four individuals, respectively. E (epithelium), LP (*lamina propria*), M (muscle), MP (*myenteric plexus*).

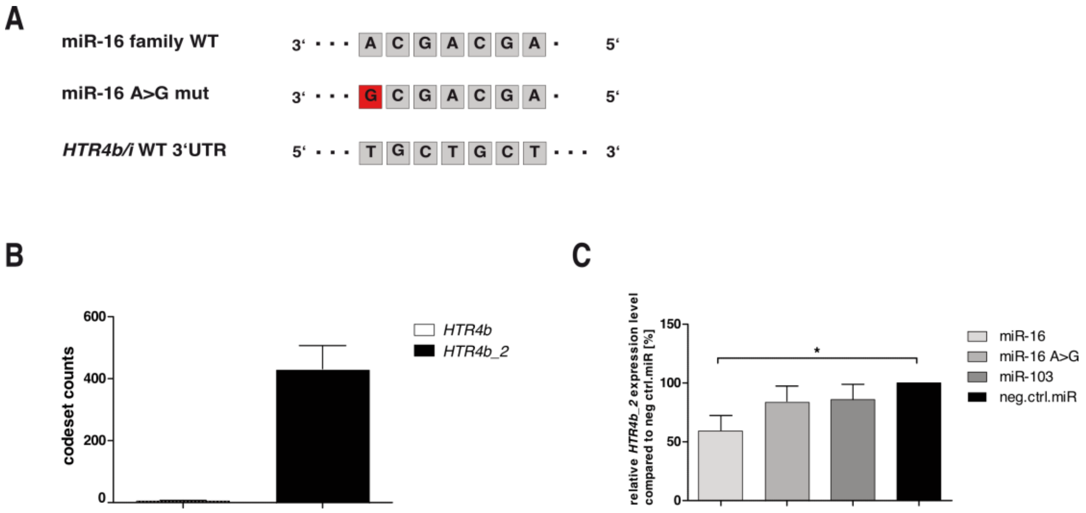

Supplementary Figure S3. Examination of *HTR4b_2* expression levels after overexpression of miRNA precursor molecules in goblet cells analysed by the nCounter technology. A) Schematic illustration of the miR-16 seed sequence wild type (WT) and mutated (mut) mimicking the rare variant found to be associated with IBS-D. **B)** Endogenous expression levels presented in codeset counts of *HTR4b* and *HTR4b_2* in

human colonic goblet cells investigated by nCounter. **C)** Comparative expression analysis of *HTR4b_2* in goblet cells transfected with miRNAs (miR-16, miR-16 A>G, miR-103) and a negative control miRNA (neg. ctrl. miR). Values are means ± SEM of four independent experiments (biological replicates). *p<0.05. Unpaired t-test.

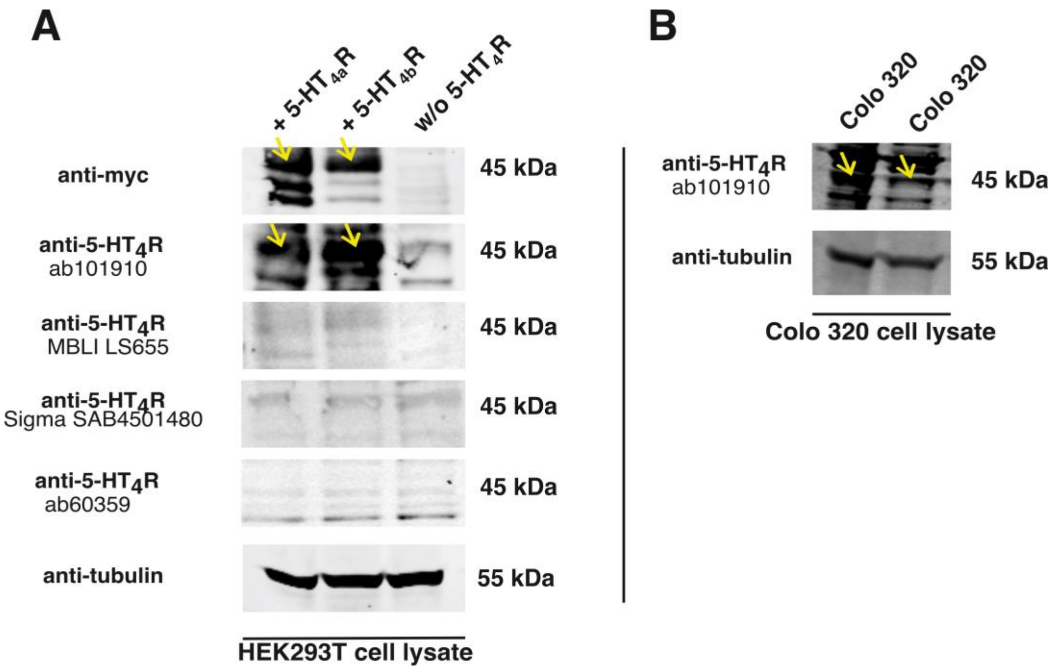

Supplementary Figure S4. Anti-5-HT$_4$R antibody characterisation in Western blot experiments. A) HEK293T cell lysates, either transfected with a myc-5-HT4a, a myc-5-HT4b or without (w/o) receptor expression construct were separated by SDS-PAGE and blotted onto a PVDF membrane. Membranes were probed with antibodies indicated to analyse their ability to specifically detect a 5-HT4 receptor protein. β-tubulin was detected to serve as loading control. **B)** Colo320 cell lysates were separated by SDS-PAGE and blotted onto a PVDF membrane. The membrane was probed with the ab101910 5-HT$_4$ receptor antibody from Abcam. β-tubulin was detected to serve as loading control.

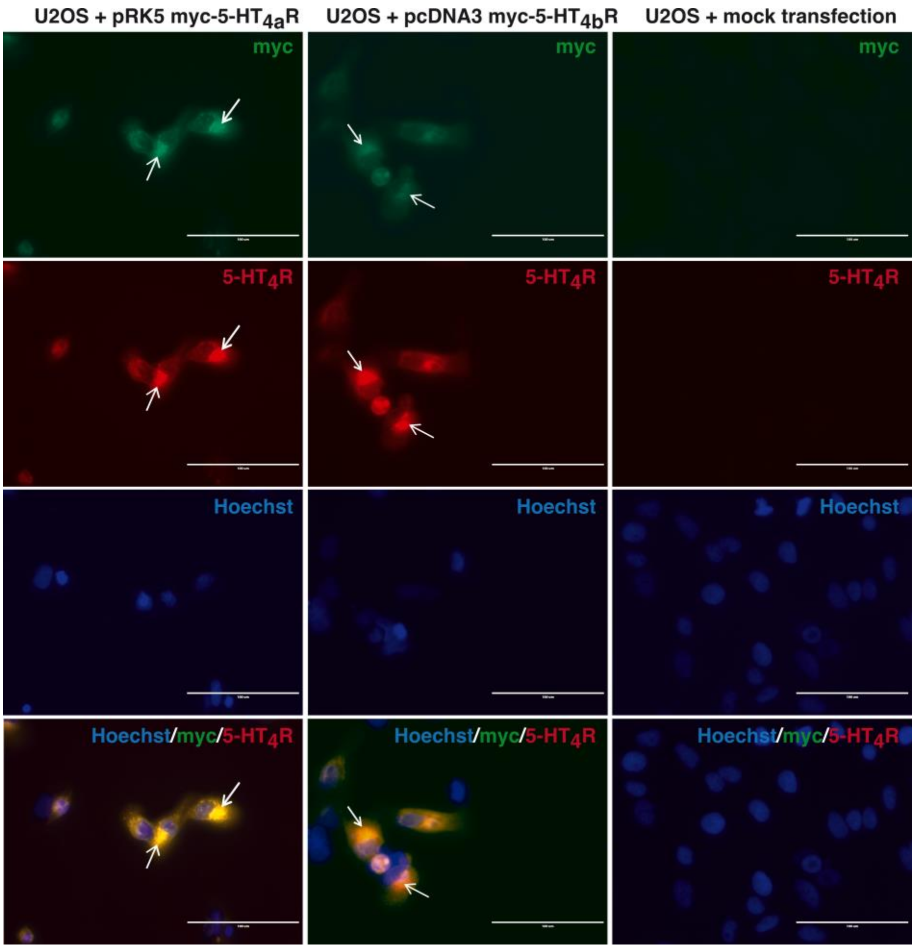

Supplementary Figure S5. Immunofluorescence characterisation of the immunoreactivity of the 5-HT$_{4b}$ receptor antibody from Sigma-Aldrich (HPA040591) with myc-5-HT4aR and myc-5_HT4bR transfected U2OS cells. Red staining (AF568) represents 5-HT4 receptor immunoreactivity. Green colour (AF488) refers to immunoreactivity of the N-terminal receptor Myc-tag. Blue staining marks nuclei (Hoechst 33342). Arrows indicate transfected cells co-stained for the 5-HT4 receptor and the Myc-tag. Scale bar: 100 µm.

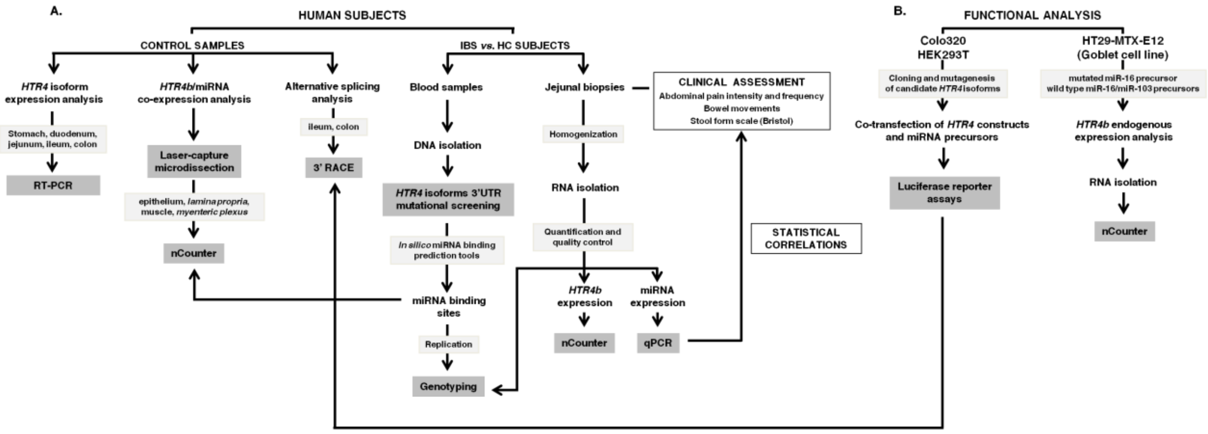

Supplementary Figure S6. Summary of experimental design. A) Workflow of the analysis done in human subjects. **B)** Functional analyses done in cell models. HC (healthy controls).
